# Supplementary figures and images for: Permissive lung neutrophils facilitate tuberculosis immunopathogenesis in male phagocyte NADPH oxidase-deficient mice
Source: PLoS Pathog. 2024 Aug 23;20(8):e1012500. doi: 10.1371/journal.ppat.1012500 (PMC11376565; doi:10.1371/journal.ppat.1012500)

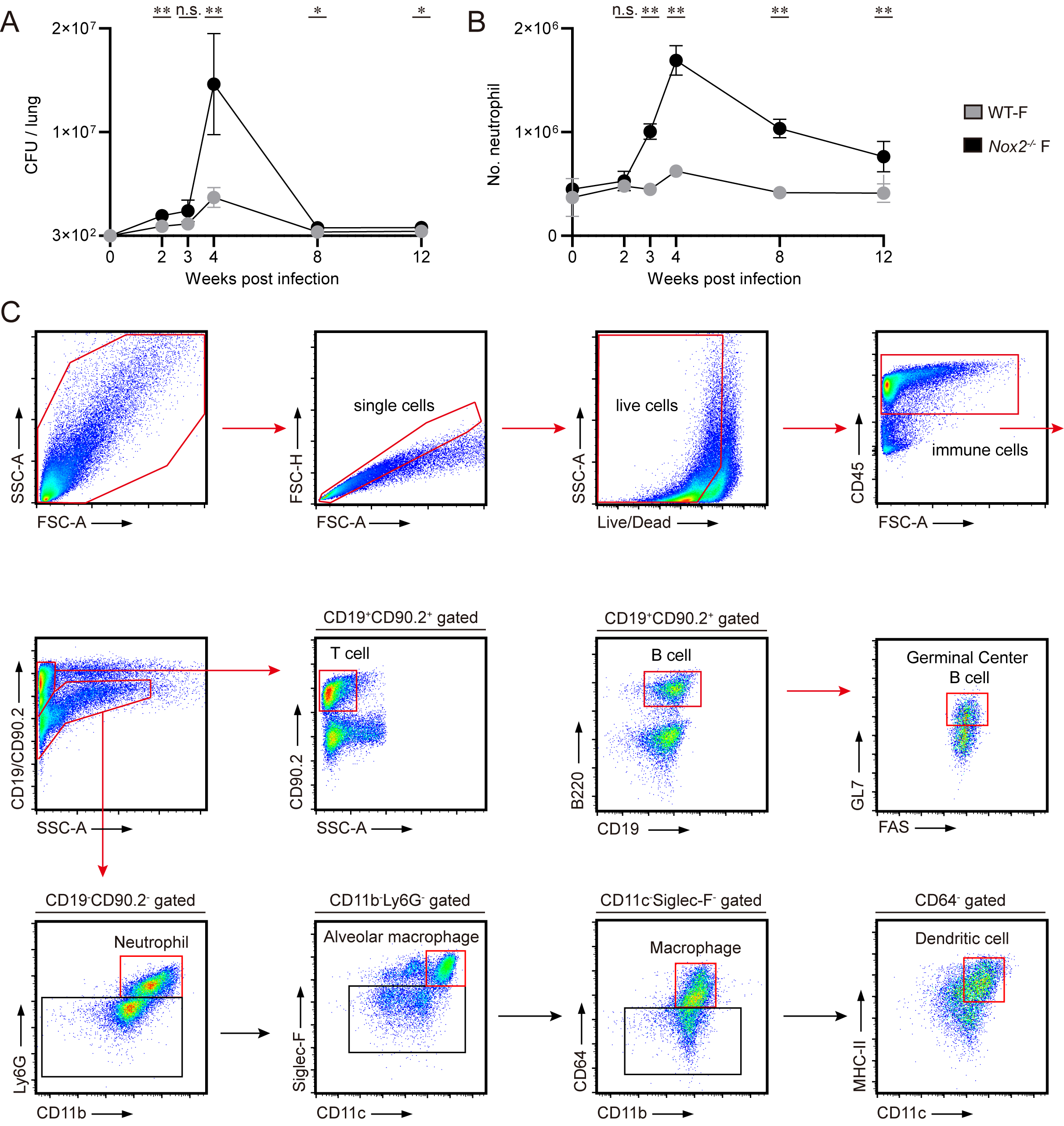

Supplement: S1 Fig — (A) Pulmonary CFUs of female WT and Nox2-/- mice were enumerated in a time-course dependent manner. six-week old female WT and Nox2-/- mice (n = 5 per group) were aerosol infected with Mtb K strain. Mycobacterial CFUs in the lungs of Mtb infected mice were calculated at 0, 2, 3, 4, 8, and 12 weeks post-infection. Initial CFU = 373. The experiment was conducted once. (B) Pulmonary neutrophil counts were calculated at 0, 2, 3, 4, 8, and 12 weeks post-infection. The experiment was conducted once. The significance of differences was determined, using the One-way ANOVA and Mann-Whitney U test. n.s., not significant. *p < 0.05. **p < 0.01. (C) The gating strategy for immune cell populations is presented as flow cytometry plots. Firstly, Live cells stained with the LIVE/DEAD Aqua Dead Cell Stain Kit were gated among single cells. CD45+ immune cells were then gated in order to investigate lung immune cell compositions. Among CD45+ immune cells, CD19+CD90.2+ SSC-Alo lymphocytes and CD19-CD90.2-SSC-Ahi myeloid cells were discriminated. Lymphocytes consisted of CD90.2+ T cells, CD19+B220+ B cells, and CD19+B220+GL7+FAS+ germinal center B cells. Myeloid cells consisted of CD11b+Ly6G+ neutrophils, CD11c+Siglec-F+ alveolar macrophages, CD11b+CD64+ macrophages, and CD11c+MHC-II+ dendritic cells. (TIF) [file ppat.1012500.s001.tif]

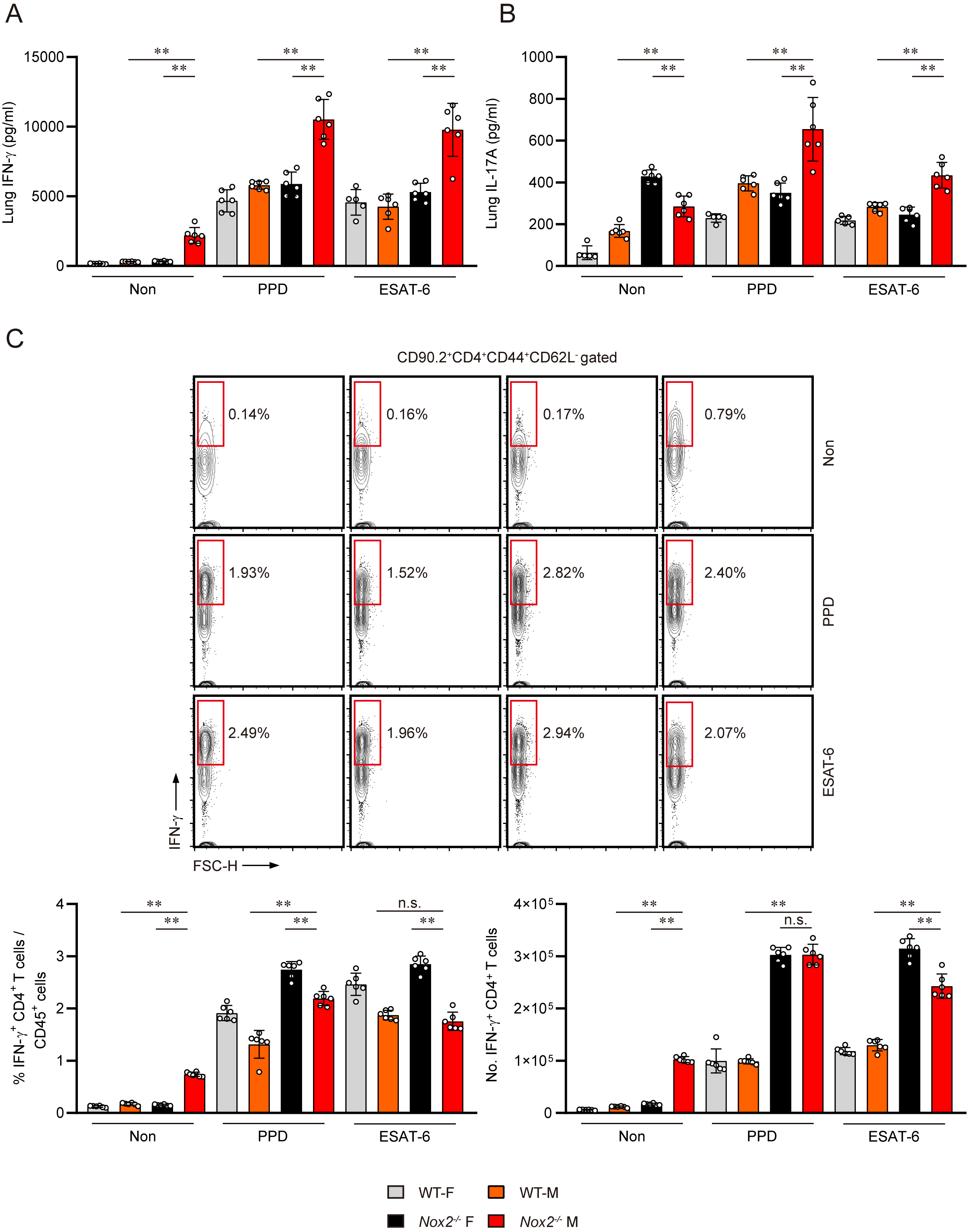

Supplement: S2 Fig — Live lung cell suspensions were cultured with or without the mycobacterial antigens ESAT-6 and PPD for 8 hours. (A) Lung IFN-γ levels and (B) Lung IL-17A levels after mycobacterial antigen stimulus are presented in bar graphs. (C) IFN-γ positive CD90.2+ CD4+ CD44+ CD62L+ effecter T cell populations are presented in bar graphs and flow cytometry plots. The data are presented as the mean ± SD of six mice in each group. The significance of differences was determined, using the One-way ANOVA test. n.s., not significant. **p <0.01. (TIF) [file ppat.1012500.s002.tif]

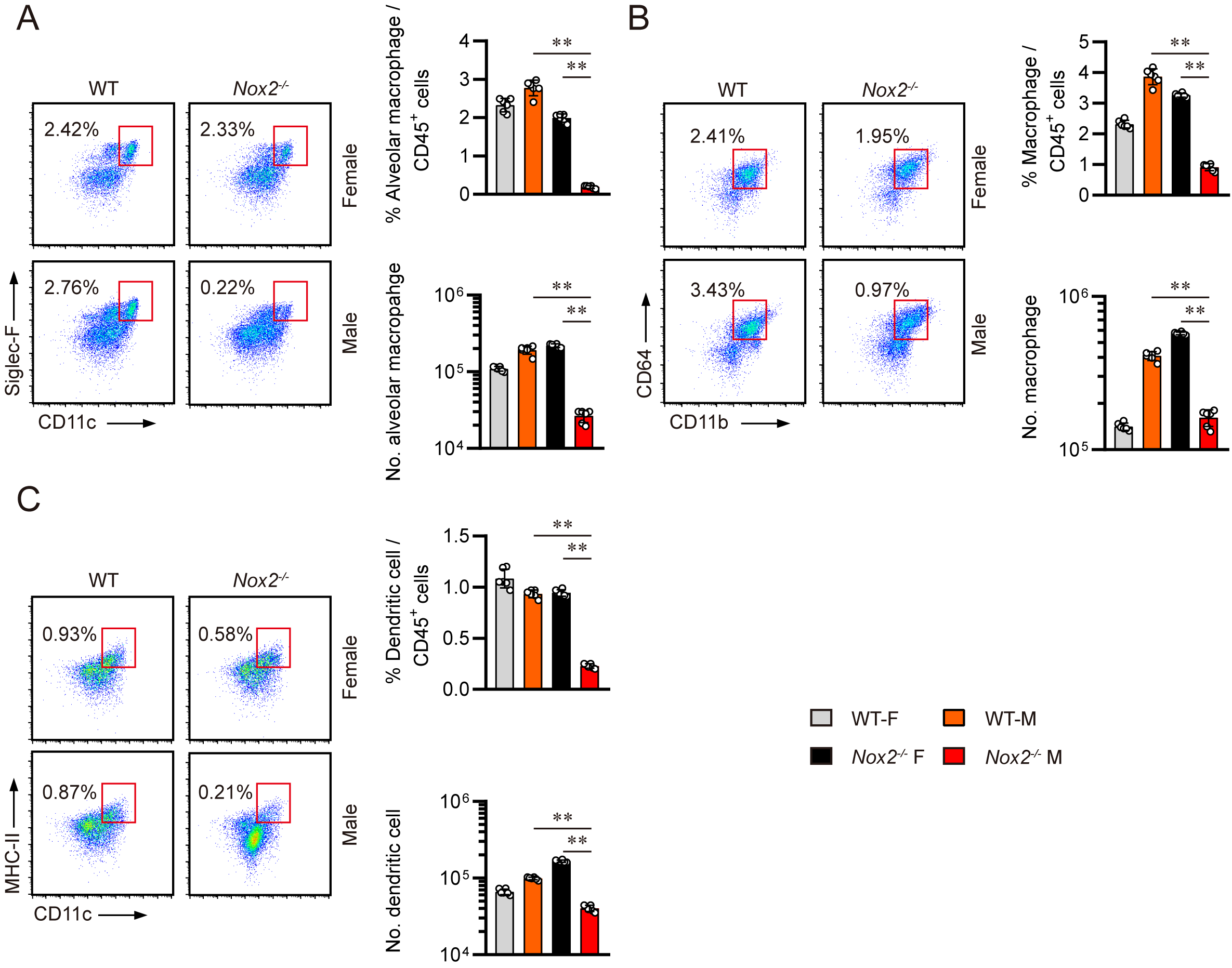

Supplement: S3 Fig — Pulmonary (A) CD11c+Siglec-F+ alveolar macrophages, (B) CD11b+CD64+ macrophages, and (C) CD11c+MHCII+ dendritic cell populations of Mtb-infected mice at four weeks post-infection. The percentages of each immune cell among lung CD45+ cells and total cell counts are presented in bar graphs. The data are presented as the mean ± SD of six mice in each group. The significance of differences was determined, using the One-way ANOVA test. **p <0.01. (TIF) [file ppat.1012500.s003.tif]

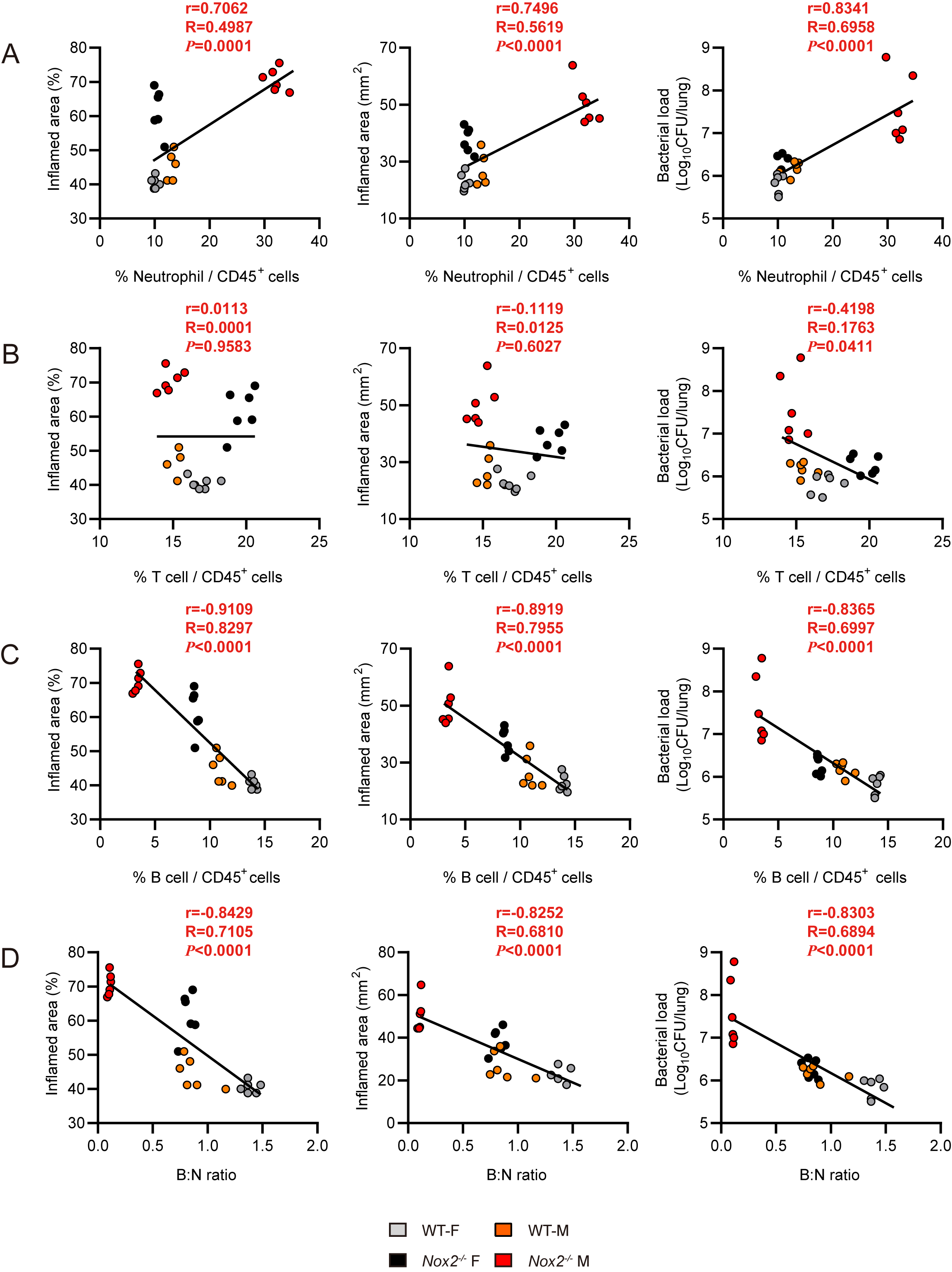

Supplement: S4 Fig — The percentages of (A) neutrophils, (B) T cells, and (C) B cells among lung CD45+ cells were individually correlated with inflamed lung percentages, inflamed lung area, and lung bacterial loads of each mouse and presented in correlation graphs. Additionally, (D) the ratio of pulmonary B cells to neutrophils (B:N ratio) was also individually correlated with inflamed lung percentages, inflamed lung area, and lung bacterial loads of each mouse and presented in correlation graphs. The significance of differences was determined by unpaired t-test and correlation analysis, featuring r and p values of each correlation. (TIF) [file ppat.1012500.s004.tif]

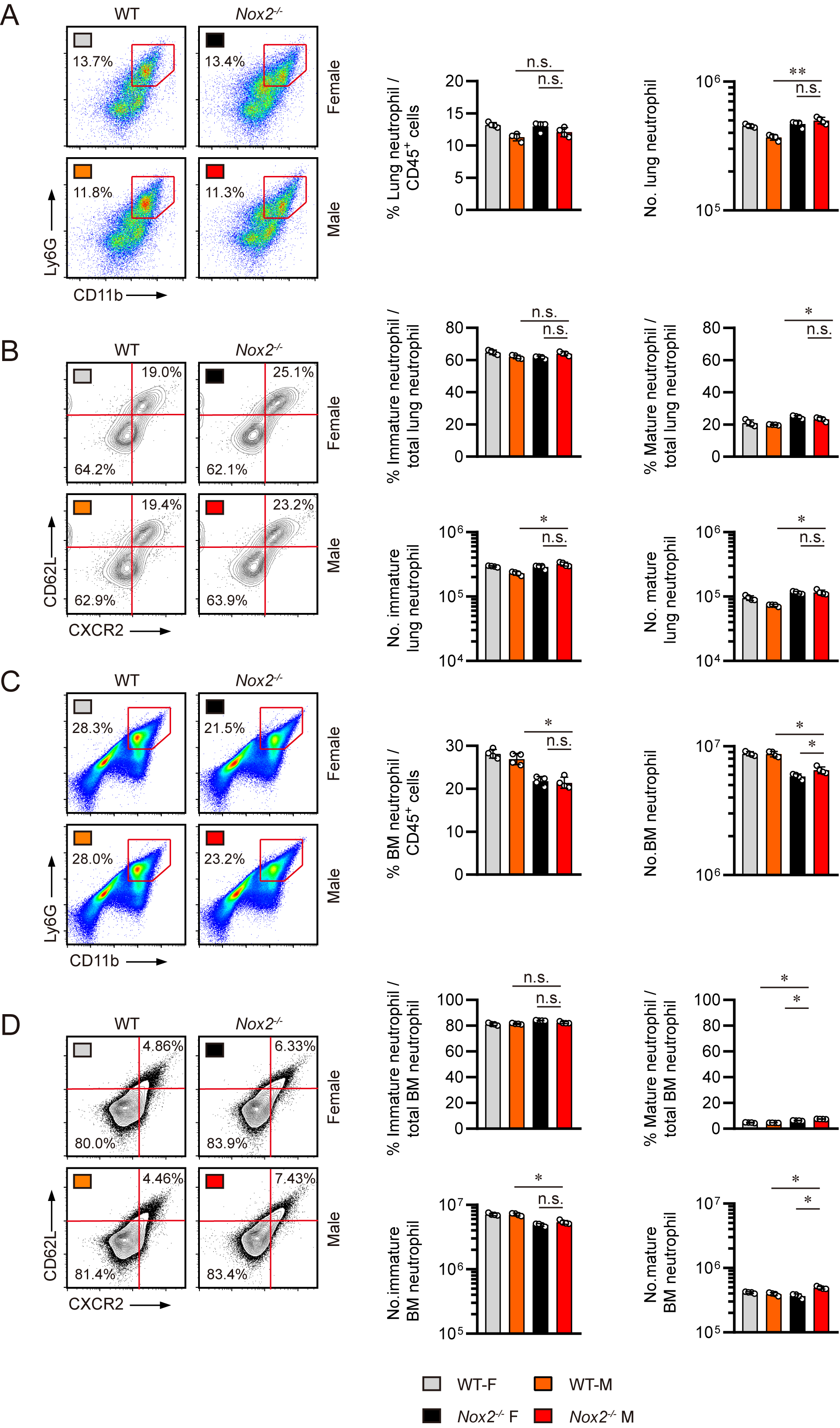

Supplement: S5 Fig — Neutrophil counts and percentages from the lungs and bone marrows of uninfected WT and Nox2-/- mice are provided (n = 4 per group). (A) The percentages of neutrophils among lung CD45+ cells and total cell counts are presented in bar graphs, along with flow cytometry plots. (B) Total cell counts and the percentages of CXCR2loCD62Llo immature neutrophils and CXCR2hiCD62Lhi mature neutrophils among total lung neutrophils are presented in bar graphs, along with flow cytometry plots. (C) The percentages of neutrophils among CD45+ bone marrow cells and total cell counts are presented in bar graphs, along with flow cytometry plots. (D) Total cell counts and the percentages of CXCR2loCD62Llo immature neutrophils and CXCR2hiCD62Lhi mature neutrophils among total bone marrow neutrophils are presented in bar graphs, along with flow cytometry plots. The data are presented as the mean ± SD of four mice in each group. The significance of differences was determined using the One-way ANOVA test and Mann-Whitney-U test. n.s., not significant. *p < 0.05. **p < 0.01. significant. (TIF) [file ppat.1012500.s005.tif]

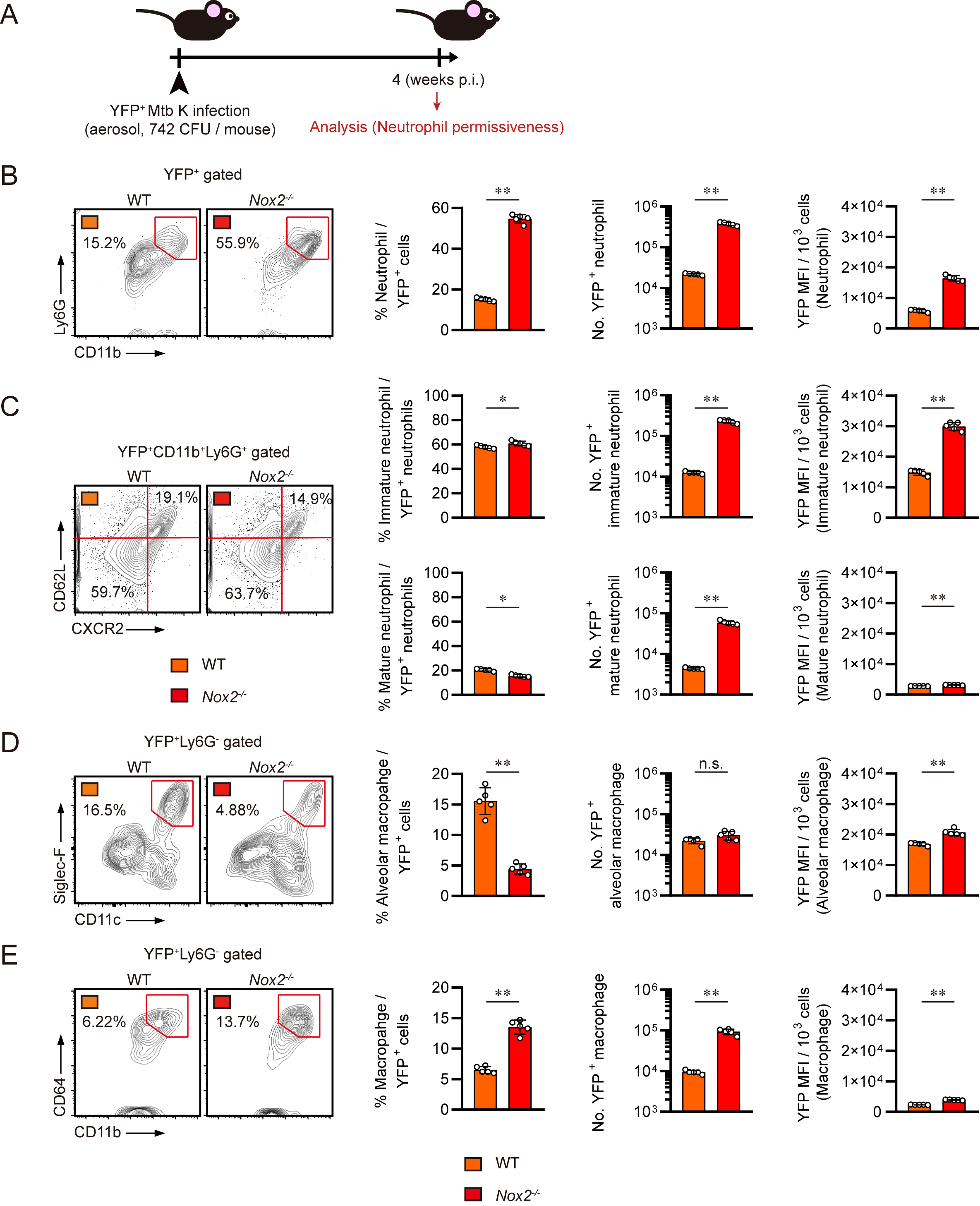

Supplement: S6 Fig — (A) Experimental design for the in vivo enumeration of permissiveness in phagocytes. Male WT and Nox2-/- mice (n = 5 per group) were aerosol infected with YFP-expressing Mtb K strain. At four weeks post-infection, all mice were autopsied, and permissiveness of neutrophils were analysed via flow cytometry (indicated by red arrow). Initial CFU = 742. (B) The percentage of neutrophils among YFP+ cells are presented in bar graphs, along with flow cytometry plots. The number of YFP+ neutrophils and MFI values of YFP in concatenated neutrophils (103 cells) are presented in bar graphs. (C) Total cell counts, percentages among YFP+ neutrophils, and MFI values of YFP (per 103 concatenated cells) in CXCR2loCD62Llo immature neutrophils and CXCR2hiCD62Lhi mature neutrophils are presented in bar graphs, along with flow cytometry plots. Total cell counts, percentages among YFP+ cells, and MFI values of YFP (per 103 concatenated cells) in (D) CD11c+Siglec-F+ alveolar macrophages and (E) CD11b+CD64+ recruited macrophages are presented in bar graphs, along with flow cytometry plots. The experiment was conducted once. The data are presented as the mean ± SD of five mice in each group. The significance of differences was determined using the One-way ANOVA test and Mann-Whitney-U test. n.s., not significant. *p < 0.05. **p < 0.01. (TIF) [file ppat.1012500.s006.tif]

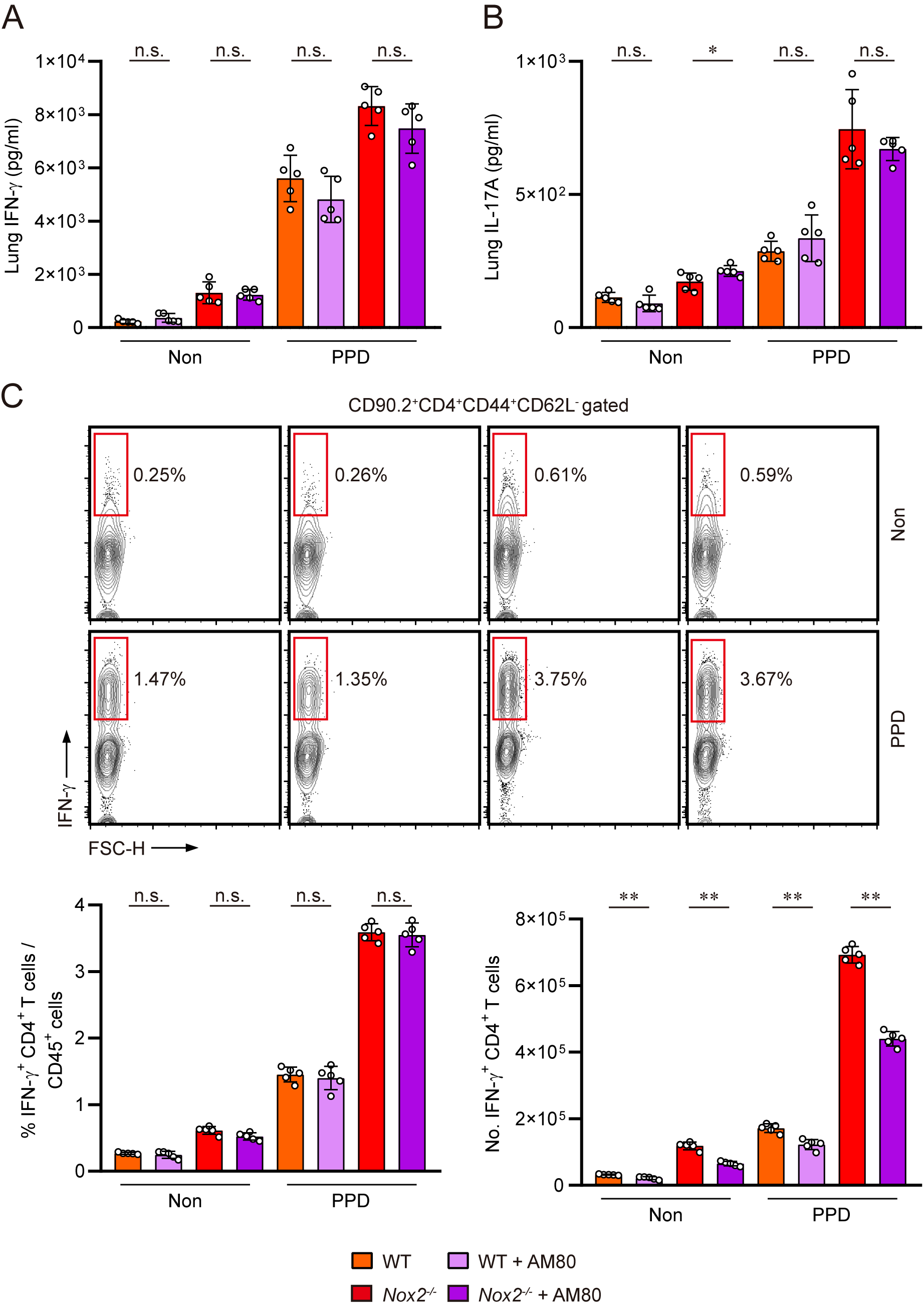

Supplement: S7 Fig — Live lung cell suspensions were cultured with or without mycobacterial antigens ESAT-6 and PPD for 8 hours. (A) Lung IFN-γ levels and (B) Lung IL-17A levels after mycobacterial antigen stimulus are presented in bar graphs. (C) IFN-γ positive CD90.2+ CD4+ CD44+ CD62L+ effecter T cell populations are presented in bar graphs and flow cytometry plots. The data are presented as the mean ± SD of five mice in each group. The significance of differences was determined, using the One-way ANOVA test and Mann-Whitney test. n.s., not significant. *p < 0.05. **p < 0.01. (TIF) [file ppat.1012500.s007.tif]

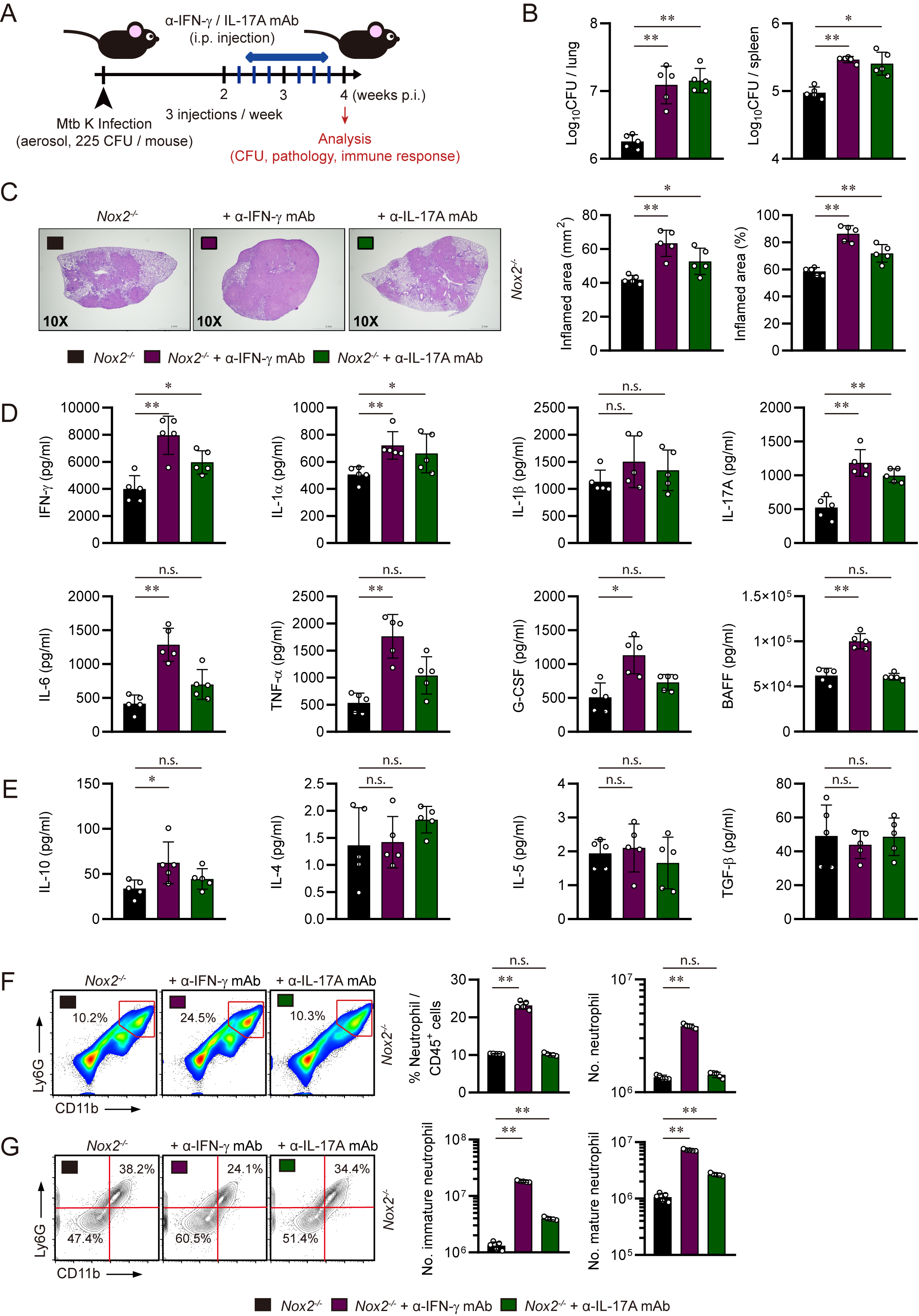

Supplement: S8 Fig — (A) Experimental design for in vivo neutralization of IFN-γ and IL-17A in Mtb infected mice. six-week old female Nox2-/- mice (n = 5 per group) were aerosol infected with Mtb K strain. Starting from two weeks post-infection, 200 μg of anti-IFN-γ mAb or 250 μg of anti-IL-17A mAb was intraperitoneally administered to each mouse three times a week (indicated by blue bars). At four weeks post-infection, all mice were autopsied, and immunological analysis, bacterial counting, and histopathological analysis were conducted (indicated by red arrow). Initial CFU = 225. (B) Mycobacterial CFUs in the lungs and spleens of each group at four weeks post-infection were analyzed by calculating the number of colonies and presented in bar graphs. (C) H&E staining was performed on the superior lobes of the right lung at four weeks post-infection to visualize the gross lung pathology. The inflamed area of the H&E-stained samples was quantified in terms of percentage and square millimeters and presented in bar graphs. (D) IFN-γ, IL-1α, IL-1β, IL-17A, IL-6, TNF-α, G-CSF, and BAFF levels in Mtb-infected mouse lung lysates were measured by ELISA and LEGENDplex (E) IL-10, IL-4, IL-5, and TGF-β levels in Mtb-infected mouse lung lysates were measured by ELISA and LEGENDplex The cytokine levels are presented in bar graphs. Pulmonary (F) CD11b+Ly6G+ neutrophil, (G) CD11b+Ly6G+CXCR2loCD62Llo immature neutrophil, and CD11b+Ly6G+CXCR2hiCD62Lhi mature neutrophil populations of Mtb-infected mice at four weeks post-infection. The percentages of each immune cell among lung CD45+ cells and total cell counts are presented in bar graphs, along with flow cytometry plots. The experiment was conducted once. The data are presented as the mean ± SD of five mice in each group. The significance of differences was determined, using the One-way ANOVA test. n.s., not significant. *p < 0.05. **p < 0.01. (TIF) [file ppat.1012500.s008.tif]

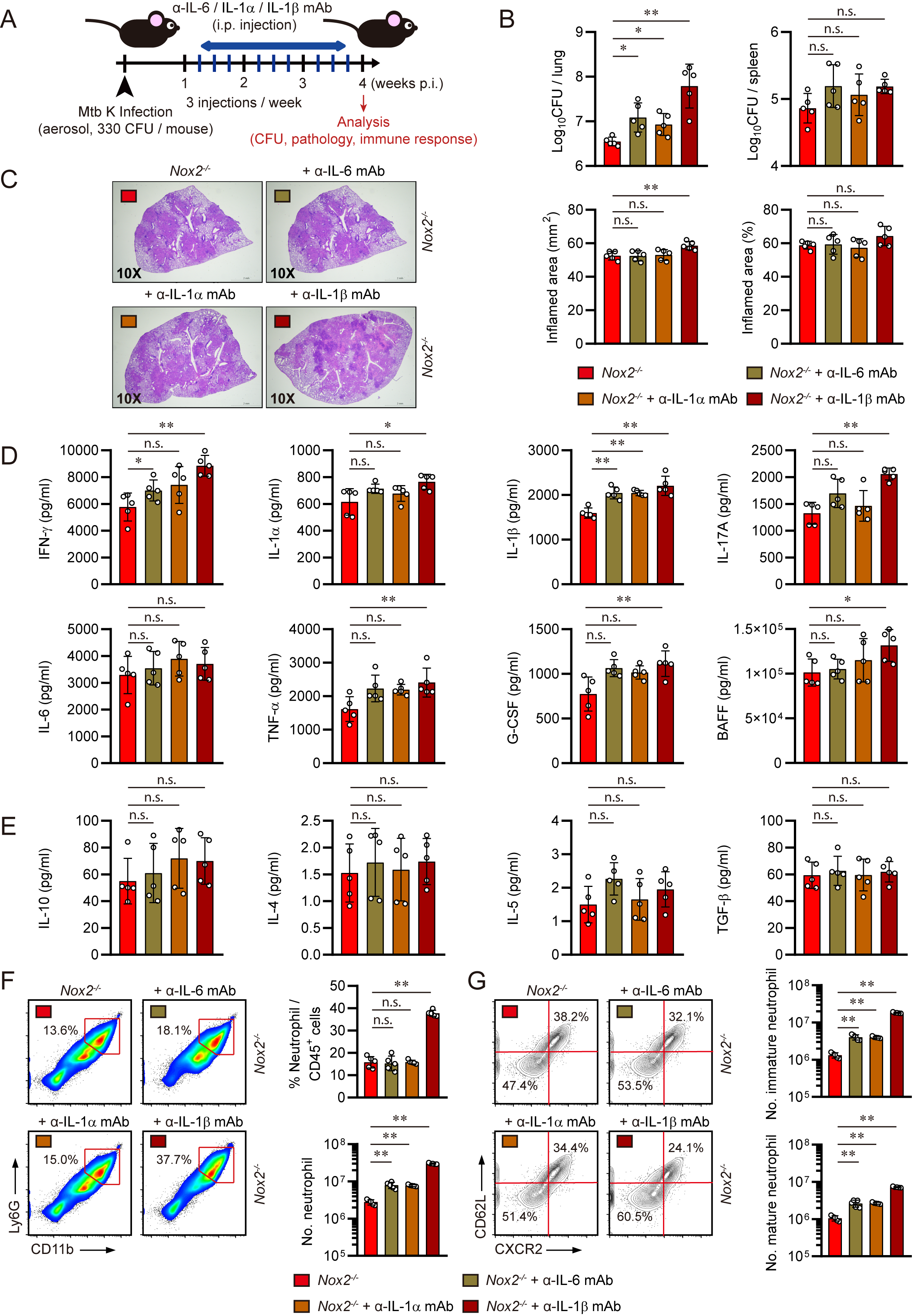

Supplement: S9 Fig — (A) Experimental design for in vivo neutralization of IL-6, IL-1α, and IL-1β in Mtb infected mice. six-week old male Nox2-/- mice (n = 5 per group) were aerosol infected with Mtb K strain. Starting from one week post-infection, 400 μg of anti-IL-6 mAb or 200 μg of anti-IL-1α mAb or 200 μg of anti-IL-1β mAb was intraperitoneally administered to each mouse three times a week (indicated by blue bars). At four weeks post-infection, all mice were autopsied, and immunological analysis, bacterial counting, and histopathological analysis were conducted (indicated by red arrow). Initial CFU = 330. (B) Mycobacterial CFUs in the lungs and spleens of each group at four weeks post-infection were analyzed by calculating the number of colonies and presented in bar graphs. (C) H&E staining was performed on the superior lobes of the right lung at four weeks post-infection to visualize the gross lung pathology. The inflamed area of the H&E-stained samples was quantified in terms of percentage and square millimeters and presented in bar graphs. (D) IFN-γ, IL-1α, IL-1β, IL-17A, IL-6, TNF-α, G-CSF, and BAFF levels in Mtb-infected mouse lung lysates were measured by ELISA and LEGENDplex (E) IL-10, IL-4, IL-5, and TGF-β levels in Mtb-infected mouse lung lysates were measured by ELISA and LEGENDplex The cytokine levels are presented in bar graphs. Pulmonary (F) CD11b+Ly6G+ neutrophil, (G) CD11b+Ly6G+CXCR2loCD62Llo immature neutrophil, and CD11b+Ly6G+CXCR2hiCD62Lhi mature neutrophil populations of Mtb-infected mice at four weeks post-infection. The percentages of each immune cell among lung CD45+ cells and total cell counts are presented in bar graphs, along with flow cytometry plots. The experiment was conducted once. The data are presented as the mean ± SD of five mice in each group. The significance of differences was determined, using the One-way ANOVA test. n.s., not significant. *p < 0.05. **p < 0.01. (TIF) [file ppat.1012500.s009.tif]

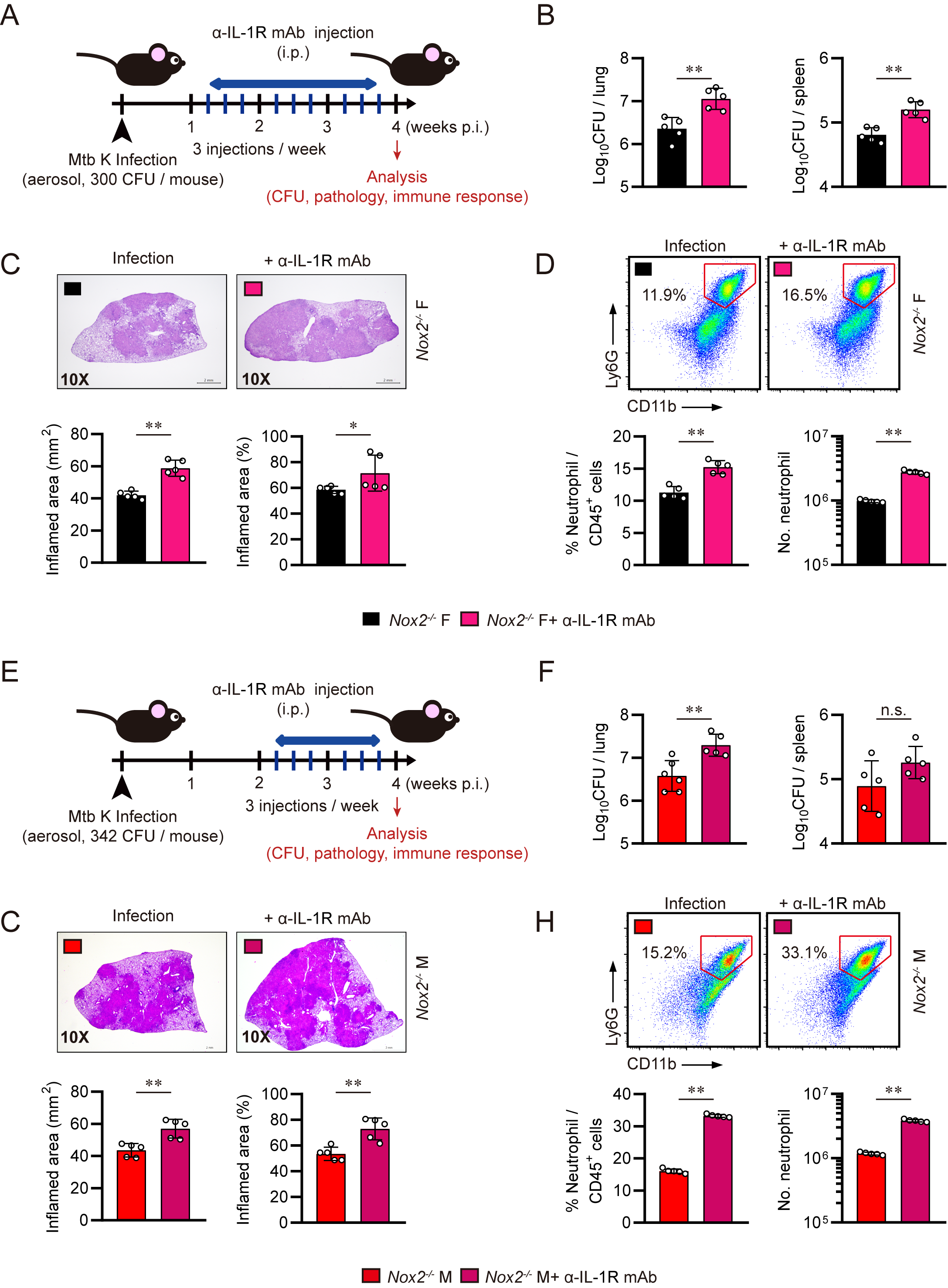

Supplement: S10 Fig — (A) Experimental design for in vivo blockade of IL-1R in Mtb infected female Nox2-/- mice. six-week old female Nox2-/- mice (n = 5 per group) were aerosol infected with Mtb K strain. Starting from one week post-infection, 200 μg of anti-IL-1R mAb was intraperitoneally administered to each mouse three times a week (indicated by blue bars). At four weeks post-infection, all mice were autopsied, and bacterial counting and histopathological analysis were conducted (indicated by red arrow). Initial CFU = 300. (B) Mycobacterial CFUs in the lungs and spleens of each group at four weeks post-infection were analyzed by calculating the number of colonies and presented in bar graphs. (C) H&E staining was performed on the superior lobes of the right lung at four weeks post-infection to visualize the gross lung pathology. The inflamed area of the H&E-stained samples was quantified in terms of percentage and square millimeters and presented in bar graphs. (D) Pulmonary CD11b+Ly6G+ neutrophil populations of Mtb-infected mice at four weeks post-infection. The percentages of neutrophils among lung CD45+ cells and total cell counts are presented in bar graphs, along with flow cytometry plots. (E) Experimental design for in vivo blockade of IL-1R in Mtb infected male Nox2-/- mice. six-week old male Nox2-/- mice (n = 5 per group) were aerosol infected with Mtb K strain. Starting from two weeks post-infection, 200 μg of anti-IL-1R mAb was intraperitoneally administered to each mouse three times a week (indicated by blue bars). At four weeks post-infection, all mice were autopsied, and bacterial counting and histopathological analysis were conducted (indicated by red arrow). Initial CFU = 342. (F) Mycobacterial CFUs in the lungs and spleens of each group at four weeks post-infection were analyzed by calculating the number of colonies and presented in bar graphs. (G) H&E staining was performed on the superior lobes of the right lung at four weeks post-infection to visualize the gross lung [file ppat.1012500.s010.tif]

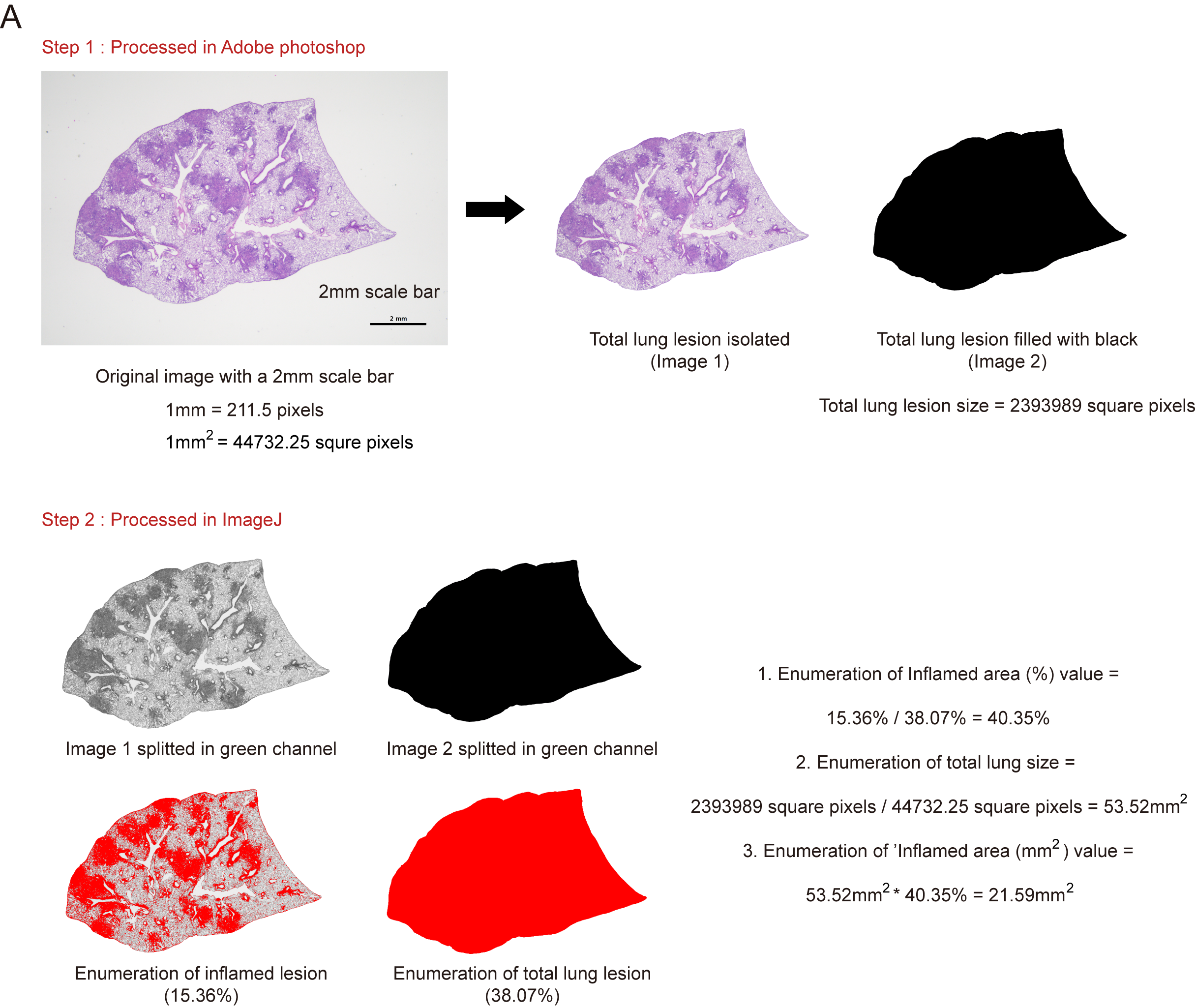

Supplement: S11 Fig — (A) As outlined in the Materials and Methods section, the inflamed area of the H&E-stained lung samples were quantified in terms of percentage and square millimetres. The values were calculated using Adobe Photoshop and ImageJ programs. New image files were created to determine the percentage of the inflamed (purple-stained) lesions, and the actual size of both total lung lesions and inflamed lesions was quantified in mm2. (TIF) [file ppat.1012500.s011.tif]
