# Supplementary material for: Permissive lung neutrophils facilitate tuberculosis immunopathogenesis in male phagocyte NADPH oxidase-deficient mice
Source: PLoS Pathog. 2024 Aug 23;20(8):e1012500. doi: 10.1371/journal.ppat.1012500 (PMC11376565; doi:10.1371/journal.ppat.1012500)
Supplement: Attachment — Submitted filename: Choi ES et al._response to reviewers.docx [file ppat.1012500.s012.docx]

**Part I - Summary**

Reviewer #1: In this manuscript “Permissive lung neutrophils facilitate tuberculosis immunopathogenesis in male phagocyte NADPH oxidase-deficient mice,” Choi et al. explore the effects of NOX2 deficiency on *Mycobacterium tuberculosis* infection. They find that NOX2 deficiency leads to increased lung inflammation and bacterial burdens, which is associated with increased inflammation and immature neutrophilia, all with a greater difference in male mice. They next go on to evaluate the potential mechanistic underpinning for these observations, showing that this susceptibility in the absence of NOX2 is in part dependent on neutrophils, and associated with immature neutrophils and G-CSF, further this can be ameliorated with a drug which stimulates the retinoic acid receptor. Overall, this study highlights a key role for NOX2 in the regulation of pathology during *Mycobacterium tuberculosis* infection and uses a diverse array of immunologic tools to characterize its effects. Enthusiasm for this manuscript is significantly tempered by three key points: 1) Even in the presence of anti-Ly6G treatment, NOX2 deficiency results in a nearly 1 log increase in CFU (fig 4B, green vs blue bars), suggesting a significant contribution of non-neutrophil factors. The authors do not address this, instead their interpretation is that neutrophils account for all of differences seen in NOX2 deficiency; 2) Related to this, NOX2 deficiency has a significant effect on other phagocytes, including macrophages, which are examined only superficially; 3) At the relatively late time point evaluated, there are already significant differences in bacterial burden and it is difficult to discern cause versus effect. This caveat could be mitigated by examining earlier time points. Lastly, it is not clear whether any of the experiments were repeated, which is critical to evaluate these findings, especially in light of the difference of these findings with published tuberculosis literature on *gp91^-/-^* mice (ref 29 in this publication), which as the authors state could be due to strain differences.

Response: Thank you sincerely for your insightful feedback on our study. By integrating your valuable advice throughout our research, we have enhanced the paper's structure. Our responses to your three main inquiries are summarized below, reiterated in parts II and III. Initially, we confirmed that NOX2 deficiency affects macrophage functions by impairing the phagocytic capacity of phagocytes in general. However, we chose to focus on the altered function and counts of neutrophils, given their prominence among immune cells in the lungs of *Nox2^-/-^* mice after Mtb exposure. The observed 1-log increase in CFU of neutrophil-depleted *Nox2^-/-^* mice is attributed to intraperitoneal neutrophil depletion, which effectively reduces circulating neutrophils but does not adequately suppress pulmonary neutrophil accumulation. This is supported by Figure 5A, showing a nearly 1-log difference in pulmonary neutrophil counts between anti-Ly6G antibody-treated WT and *Nox2^-/-^* mice. Furthermore, we examined the time-dependent impacts of NOX2 deficiency in female mice before investigating and comparing sexual variations in *Nox2^-/-^* mice. Time-point-focused studies revealed that *Nox2^-/-^* mice do not exhibit neutrophilia and increased bacterial load at early time points (2W and 3W post-infection), with differences peaking at 4W post-infection. Interestingly, the pathological gap between WT and *Nox2^-/-^* mice decreased after 4W post-infection, persisting until the 12W post-infection period. Regarding interventions, positive interventions (neutrophil depletion, AM80 administration, and G-CSF neutralization) were repeated at least twice. However, negative interventions (IL-1α neutralization, IL-1β neutralization, IL-6 neutralization, IL-17A neutralization, IFN-γ neutralization, and IL-1R blockade) were conducted only once.

Reviewer #2: In this manuscript by Choi et al the investigators examine differences in role of phagocyte oxidase mediated protection in male and female mice. They find that male *Nox2^-/-^* mice have higher bacterial burden following infection with a virulent Strain K, while female mice do not. They find increased immature neutrophil recruitment and lung damage at 4 weeks post-infection that is associated with higher Mtb levels and a hyperinflammatory response. Depleting neutrophils in male mice reversed the increased CFU and hyperinflammation while IL-1 blockade did not. In contrast, treatment with AM80 and anti-G-CSF lowered bacterial levels in male *Nox2^-/-^* mice and reversed the inflammatory state. The authors conclude that increased G-CSF in male mice specifically results in the recruitment of immature neutrophils that drive inflammation and increased bacterial burden. The paper is generally clear and the experiments are mostly well controlled with a few caveats. Several of the key observations including the role of increased neutrophil recruitment and the role of immature neutrophils in driving disease have previously been described. Furthermore, several of the data presented appear to conflict with other previous reports using various knockouts of distinct subunits of the NADPH phagocyte oxidase, yet the reasons for these discrepancies are not fully understood/examined. Without further experiments or controls this leaves some of the conclusions not fully supported by the data as is.

Response: Thank you greatly for your valuable comments and insights. Other studies have explored the involvement of immature neutrophils in lung diseases, but our group was the pioneering one to identify the G-CSF-mediated rise in pulmonary neutrophils during Mtb infection. Moreover, our investigation into the therapeutic potential of AM80 administration and G-CSF neutralization in *Nox2^-/-^* mice further underscores the novelty of our research. The utilization of the highly virulent Mtb K strain and the male *Nox2^-/-^* mouse model reinforces the novelty of our paper. Specifically, we shed light on biological sex-dependent variations in TB susceptibility in *Nox2^-/-^* mice, which correlates notably with the generation of immature neutrophil subpopulations induced by G-CSF. In parts II and III, we will delve into supplementary data discussed in the revised sections.

Reviewer #3: Choi and colleagues in this study have characterized the lung neutrophils phenotype in female and male Mtb infected wild type (WT) and NOX2KO mice; and showed that increased immature CXCR2^lo^CD62L^lo^ Ly6G^+^ lung neutrophils were associated with enhanced TB pathology in male NOX2KO mice. They performed depletion experiments using antibodies against various cytokines and found that G-CSF-driven differentiation of immature neutrophils was responsible for exacerbated TB pathology in male NOX2KO mice. These kinds of studies are important to dissect the pathologic and protective role of neutrophils in TB and possible in other infectious diseases. What is the phenotype of depleting immature neutrophils in WT mice? Does depleting G-CSF in WT mice has an effect on TB pathology? These biological questions and significant technical issues lowered the interest of this reviewer.

Response: Your sincere and insightful feedback on our research is greatly appreciated. In response to your comments, we have included *in vivo* data on the effects of AM80 administration and G-CSF neutralization in WT mice. While neutrophil depletion led to a slight reduction in TB progression in WT mice, the loss of immature neutrophils induced by AM80 and G-CSF neutralization did not significantly impact the pathogenesis of WT mice. From these findings, we hypothesize that neutrophils play a generally detrimental role in the TB pathogenesis of mice, whereas excessive G-CSF-mediated immature neutrophils are specifically critical for the pathogenesis of NOX2KO mice. This underscores the significance of excessive G-CSF-driven TB pathogenesis in male *Nox2^-/-^* mice.

**Part II – Major Issues: Key Experiments Required for Acceptance**

Reviewer #1:

Given the large differences in bacterial burdens, it is difficult to know which changes in immune cell number/proportion and increased inflammatory cytokines seen in the male NOX2 deficient mice are reflective of increased bacterial burdens, or responsible for driving the pathology. To gain better insight into the mechanistic determinants for NOX2-driven pathology, the authors could evaluate responses at an earlier timepoint, when there is no (or less) discrepancy in bacterial burdens. Also, why is this data different from the published *gp91^-/-^*, as above do the authors think this is solely related to bacterial strain, or potentially could infectious dose also influence these responses?

[A1]: Thank you very much for your feedback on the data. Prior to conducting experiments on male and female mice, we conducted a time-course experiment comparing mycobacterial CFUs and neutrophilia using female WT and *Nox2^-/-^* mice. Our observations indicate that pulmonary mycobacterial load and neutrophilia did not show significant differences in *Nox2^-/-^* mice compared to WT mice at 2 weeks post-infection. However, the disparity between WT and *Nox2^-/-^* mice increased at 3 weeks post-infection, reaching its peak at 4 weeks post-infection. Furthermore, the disparities in lung bacterial load and neutrophil counts decreased after 4 weeks post-infection until 8 and 12 weeks post-infection. We found that the increased mycobacterial burden in *Nox2^-/-^* mice correlates with neutrophilia, which was a notable immunologic feature in Mtb infected *Nox2^-/-^* mice. We focused on the 4-week post-infection time point in all experiments, as the TB severity and neutrophilia of *Nox2^-/-^* mice were optimized at this time point.

The mice were euthanized at the four-week post-infection time point because this is when the NOX2-deficiency mediated increase in lung bacterial load (S1A Fig) and neutrophil infiltration (S1B Fig), a key feature of Mtb-infected *Nox2^-/-^* mice, were maximized.

[A2]: In studies involving male *Nox2^-/-^* mice, which exhibited enhanced susceptibility compared to female *Nox2^-/-^* mice, neutrophils were identified as the predominant immune cell type in the lungs of Mtb-infected male *Nox2^-/-^* mice at 4 weeks post-infection. Therefore, our primary objective was to investigate the properties of NOX2 deficient neutrophils, as they were identified as the most dominant immune cell type and correlated with the increased bacterial load in *Nox2^-/-^* mice. Furthermore, we discovered that these neutrophils are permissive, indicating their role in the increased pulmonary bacterial load in NOX2KO mice, which is driven by excessive G-CSF. It is noteworthy that both the mycobacterial strain and the infectious dose likely influenced TB pathogenesis in male NOX2KO mice. While Thomas et al. utilized the H37Rv strain in their experiments, we employed the Mtb K strain, a clinical isolate known for its high virulence associated with a school outbreak. Additionally, our mice were infected with a high dose of CFUs, more than 200 CFUs per mouse. Initial mycobacterial load did not differ significantly between WT and *Nox2^-/-^* mice. Hence, the utilization of the highly virulent Mtb K strain and the high CFUs likely contributed to the exacerbated TB progression observed in our studies.

The methods outlined for determining extent of inflammation are not sufficient to understand what this is measuring. The listed reference also does not have enough detail (overall there is not enough detail in the methods). In the text, authors refer to “necrotic inflammation” whereas the figure axes are labeled “inflamed area,” which are quite different. Also, from thin tissue sections of a region of interest, I’m not sure how useful the absolute area of inflammation is, the percent inflammation controls for the size of the section, which is the most relevant to analyze. Overall, the images aren’t at a sufficient resolution to evaluate in the uploaded tif files. Perhaps the authors could include images from all evaluated animals in a supplemental figure?
[A3]: We added additional methods for enumerating inflamed area of lung. We also considered that calculating inflamed area only through H&E staining would be insufficient. Therefore, we added up cytokine profiles from lung lysates for each data. Also, as TB pathogenesis in susceptible mouse models generally cause expansion of lung, we both calculated inflamed percentages (%) and sizes of inflamed area (mm2). We re-uploaded image files with high resolutions for each data.

The lung sizes were determined using Adobe Photoshop CS6 (ADOBE, San Jose, California) by comparing the pixel values of size bars and lung lobes. Inflamed lung percentages (%) were calculated by counting inflamed lung lesions across the entire lung area using ImageJ. The sizes of inflamed areas (mm2) were computed by multiplying the percentages of inflamed lung by the total lung section sizes for each mouse.

The authors propose that immature lung neutrophils are permissive, but never show this specifically on immature neutrophils in the setting of NOX2 deficiency. In an above (or related) experiment this could be shown by sorting and plating immature and mature neutrophils. Otherwise, the authors should make a statement more generally about neutrophils, which they have shown have higher bacterial burdens.

[A4]: We conducted an additional *in vivo* experiment to enumerate the mycobacterial permissiveness of immature neutrophils by challenging with the YFP-expressing Mtb K strain. YFP-expressing vectors were generously provided by Dr. Christopher Sasetti. Following four weeks of challenge with the YFP-expressing Mtb K strain, we observed that CD11b^int^Ly6G^int^ CXCR2^lo^ CD62L^lo^ immature neutrophils constituted the majority of YFP+ cells in the lungs of male *Nox2^-/-^* mice. Furthermore, we found that these immature neutrophils exhibited higher YFP MFI values compared to mature neutrophils, indicating their heightened permissiveness. Consequently, we concluded that immature *Nox2^-/-^* neutrophils are highly permissive, which plays a crucial role in increasing the pulmonary bacterial load in male *Nox2^-/-^* mice.

To investigate whether CXCR2^lo^CD62L^lo^ immature neutrophils exhibit enhanced mycobacterial permissiveness compared to CXCR2^hi^CD62L^hi^ mature neutrophils, we infected male WT and Nox2-/- mice with YFP-expressing Mtb K strain. At four weeks post-infection, the mice were euthanized, and neutrophil permissiveness was assessed through flow cytometry analysis (S5E Fig). We observed that neutrophils comprised the majority of YFP+ cells in male *Nox2^-/-^* mice, and the number of Mtb-containing neutrophils was also increased in *Nox2^-/-^* mice. Notably, YFP MFI values of *Nox2^-/-^* lung neutrophils were significantly elevated compared to those of WT mice, suggesting heightened permissiveness (S5F Fig). Furthermore, we found that both Mtb-containing immature and mature neutrophil populations were increased in *Nox2^-/-^* mice. Crucially, immature neutrophils demonstrated markedly higher YFP MFI values than mature neutrophils, indicating their heightened permissiveness to mycobacteria (S5G Fig).

AM80 treatment has a relatively modest effect on the absolute number of neutrophils present. Given the mechanism proposed, is there a difference in the percentage of immature neutrophils? Also, the proportion of neutrophils does not seem to change, which is similar to other cell populations. Are the absolute numbers of all cell populations decreased? If so, it doesn’t seem fair to conclude that immature neutrophils are the key cell population, if many other cell populations changed as well. Given the effects of ATRA treatment in reference 58, were responses of T cells and macrophages evaluated further?

[A5]: AM80 treatment decreased both the absolute number and percentage of immature neutrophils in *Nox2^-/-^* mice. We examined T cell responses after ATRA treatment and found that ATRA treatment does not significantly alter Mtb-specific T cell responses in *Nox2^-/-^* mice. Prior to conducting AM80 treatment in in vivo challenges, we tested the effects of AM80 on *Nox2^-/-^* BMDMs but did not observe significant changes in the phagocytic capacities of BMDMs. Therefore, as AM80 did not significantly alter macrophage or T cell responses in *Nox2^-/-^* mice, we concluded that its role in promoting neutrophil maturation is crucial for mitigating TB pathogenesis in *Nox2^-/-^* mice, as it was originally developed to promote mature neutrophil generation in leukemia patients.

While AM80 treatment effectively modified the accumulation of immature lung neutrophils, it did not exhibit significant effects in promoting Mtb-specific T cell responses in WT and *Nox2^-/-^* mice (S6 Fig). This suggests that the therapeutic potential of AM80 intervention in male *Nox2^-/-^* mice is largely contingent on the conversion of neutrophil maturity.

Reviewer #2: Major Comments:

1. Several previous papers cited by the authors show that infection of Phox deficient mice does not result in major changes to CFU over time in contrast to what is shown. The authors suggest this is due to difference strains however, but this is not directly tested. The authors only examine a single time point in their studies (4 weeks post infection) and do not examine the kinetics of bacterial growth over time which were shown to have some variability during infection or the impact on survival long term. The authors use a wild type C57BL6N background which also differs from previous studies. It is unclear from the methods whether the *Nox2^-/-^* mouse is also on the C57BL6N or the C57BL6J background. This should be made clearer as a potential confounder.

[A1]: Thank you for your appreciation of the valuable comments. We decided to utilize the highly virulent Mtb K strain in our experiments with *Nox2^-/-^* mice, as this strain has demonstrated increased virulence compared to the H37Rv strain in previous *in vivo* studies conducted by our group. Before initiating experiments with male *Nox2^-/-^* mice, we conducted a preliminary assessment of bacterial growth kinetics and neutrophilia to determine the optimal time point for analysis. Our data revealed that pulmonary mycobacterial load and neutrophilia did not exhibit significant differences in *Nox2^-/-^* mice compared to WT mice at 2 weeks post-infection. However, the disparity between WT and *Nox2^-/-^* mice increased at 3 weeks post-infection, peaking at 4 weeks post-infection. Subsequently, the disparities in lung bacterial load and neutrophil counts decreased after 4 weeks post-infection, persisting until 8 and 12 weeks post-infection. Thus, given that TB progression and neutrophilia in *Nox2^-/-^* mice were optimized at 4 weeks post-infection, we focused on elucidating the immunologic factors responsible for the construction of neutrophil-mediated TB pathogenesis in male *Nox2^-/-^* mice at this specific time point.

The mice were euthanized at the four-week post-infection time point because this is when the NOX2-deficiency mediated increase in lung bacterial load (S1A Fig) and neutrophil infiltration (S1B Fig), a key feature of Mtb-infected *Nox2^-/-^* mice, were maximized.

[A2]: The *Nox2^-/-^* mice used in our studies are of C57BL6N background. We determined that the C57BL6N mouse strain is preferable as a genetic background for NOX2-deficient mice in our studies with the Mtb K strain. This decision was based on our observation that C57BL6N mice exhibit slightly more severe TB pathogenesis compared to C57BL6J mice.

2. For several of the treatment studies including AM80, G-CSF, IL1a, IL1b, IL6 there are no wild type controls to understand changes in control mice. This is particularly important for AM80 which the authors conclude is driving better neutrophil maturation and control, however, this could also be directly acting on infected macrophages to lower bacterial burden as has been shown previously in human macrophages. Without knowing if AM80 is specifically driving neutrophil mediated control these conclusions are not supported by the current data. The authors also conclude the changes in Male Nox2-/- mice are not due to IL1a or IL1b but a co-treatment is not done even though these cytokines can act redundantly. Furthermore, it is known that IL1 signaling is important for early control of Mtb infection yet depletion beings very early following aerosol infection which again, may be driving changes in macrophage bacterial levels that then amplify differences in neutrophil responses. This is not addressed through experiments.

[A3]: We incorporated results from WT mice treated with AM80 and G-CSF neutralization into our study. Our focus was on elucidating the roles of AM80 in neutrophil maturation, as it did not alter the phagocytic capacities of BMDMs and T cell responses of *Nox2^-/-^* mice. We did not perform additional studies or interventions on WT mice with IL-1a, IL-1b, and IL-6 neutralization, as these interventions were deemed unlikely to control neutrophilia based on a screening concept. Since our primary objective was to develop potential therapeutic interventions, we expanded our dataset for WT mice and replicated these experiments on AM80 treatment and G-CSF neutralization.

[A4] We treated female *Nox2^-/-^* mice with IL-1 receptor blocking monoclonal antibody, which inactivates both IL-1a and IL-1b, as it was found to be lethal in male mice. Our study revealed that IL-1R signaling exacerbates TB pathogenesis. Consequently, we found that both IL-1a and IL-1b are essential for proper defense against Mtb in NOX2KO mice. As pulmonary neutrophilia in *Nox2^-/-^* mice reaches its peak between 2 to 4 weeks post-infection, our objective was to identify immunologic factors contributing to neutrophil generation during this period. However, unlike G-CSF neutralization, neither IL-1a nor IL-1b neutralization succeeded in suppressing the explosive generation of neutrophils. Consequently, we concluded that G-CSF, rather than IL-1, is responsible for the accumulation of neutrophils between the 2 to 4-week post-infection time point.

Blockade of IL-1 receptor also exacerbated TB progression in female *Nox2^-/-^* mice (S8 Fig)

3. The majority of the statistics seem to use the Mann-Whitney U test which from my understanding is a comparison between two groups and in several experiments there are more than two comparisons being made. A more appropriate test should be used for these experiments to account for multiple comparisons. Furthermore, in the figure legends it is unclear how many replicate experiments were done for each data panel making it difficult to judge the reproducibility of the results.

[A5]: Thank you for mentioning this crucial point for the revision of our study. As you have mentioned, I have adapted the ANOVA test for comparing more than two groups. Additionally, I have indicated the number of replications in each experiment conducted.

4. The methods in general are not sufficient for reproducible studies. While previous studies can be cited the manuscript should contain enough information for readers to fully understand the results. For example, how was the Strain K cultured? Is it PDIM positive? For the aerosol infections, was the day 0 lung CFU value calculated for each experiment (if so this should be explicitly written in the figure legends)? Were there differences in these values between male and female mice that may drive downstream phenotypes? These data are important for the readers to understand all the parameters of the experiments.

[A6]: We appreciate you so much for a crucial comment for us and our fellow researchers. The methods for culturing the Mtb K strain have been updated. We have utilized the PDIM-positive Mtb K strain for every single experiment. Initial CFUs for each experiment are included in the figures and figure legends. No significant differences in initial CFUs were observed between WT and *Nox2^-/-^* mice, nor between male and female mice.

The mycobacteria were cultured in 7H9 broth supplemented with 10% oleic acid-albumin-dextrose-catalase (OADC; Difco Laboratories, MD) and 2 μg/ml of mycobactin J (Allied Monitor, Fayette, MO) for 4 weeks (slow growers) at 37°C. Following cultivation, all bacteria were washed three times with 10 mM phosphate-buffered saline (PBS; pH 7.4). Bacterial cell pellets were collected after centrifugation, and small aliquots were stored at -80°C until use. The Mtb K strain used for in vivo challenges was PDIM positive. YFP vectors for the generation of YFP+ Mtb K strain were kindly gifted by Dr. Christopher Sassetti.

**Reviewer #3:**

1. Thomas et al. 2023 have earlier demonstrated that gp91phox-deficient mice displayed enhanced inflammation and lung neutrophil infiltration after Mtb challenge, which were mediated by IL-1. In this context a Fig 1 and Fig 2 of this manuscript is just repeating what was published before. They can be merged into one figure. Having said that the current manuscript though brings the angle of sex, which is interesting.

[A1]: I would like to appreciate for your advices and comments, which will be a great help for developing our study. In figure 1 and 2, we aimed to emphasize the sexual difference between male and female *Nox2^-/-^* mice, which is a novel finding in comparison to previous studies. Our major point from figure 1 is that male *Nox2^-/-^* mice feature severer bacterial load, lung inflammation and pulmonary inflammatory cytokine expression in comparison to WT and female *Nox2^-/-^* mice. On the other hand, major point of figure 2 is that male *Nox2^-/-^* mice features altered pulmonary immune cell compositions, with extreme neutrophilia and loss of lymphocytes. While figure 1 focuses on bacterial load and inflammation, figure 2 focuses on immune cell compositions, which will be a key clue for targeting neutrophils as the major drivers of TB pathogenesis. As these two figures focus on different aspects of immune features of male *Nox2^-/-^* mice, we separated the data into two figures. Also, we tried to emphasize that sexual differences strongly contribute to TB susceptibility by comparing immune profiles of male *Nox2^-/-^* mice with WT and female *Nox2^-/-^* mice, as female *Nox2^-/-^* mice did not feature extreme neutrophilia as much as male *Nox2^-/-^* mice.

2. Immature neutrophils and pathology: Regarding the observation that NOX2KO mice exhibited higher levels of immature lung neutrophils, inflammation and mycobacterial burden compared to female NOX2KO mice and WT mice after Mtb challenge. Is this due to the inherent nature of higher immature neutrophils in the lung of mice at the time of infection, or those increased immature neutrophils arrive only upon infection (granulopoisis in bone marrow). Thus, it is important to know the neutrophil phenotype (possibly in lung and bone marrow) in uninfected female / male WT and NOX2KO mice.

[A2]: We have included a supplementary figure illustrating neutrophil populations in uninfected mice. Our analysis confirmed that there were no significant differences in neutrophil populations between uninfected male *Nox2^-/-^* mice and WT or female *Nox2^-/-^* mice. Additionally, the proportions of immature and mature neutrophils in the lungs and bone marrow of male *Nox2^-/-^* mice did not show any discrepancies. Therefore, we concluded that the extreme infiltration of pulmonary neutrophils is likely triggered by mycobacterial stimulus.

We also analyzed neutrophil profiles from uninfected WT and *Nox2^-/-^* mice. Pulmonary total and immature neutrophils (S5A, S5B Fig), as well as bone marrow (BM) (S5C, S5D Fig) total and immature neutrophils, were not increased in male *Nox2^-/-^* mice. Therefore, we concluded that the extreme pulmonary infiltration of CXCR2loCD62Llo immature neutrophils is initiated by the pathogenic stimulus triggered by Mtb infection.

3. In Fig S4 authors showed that “The loss of pulmonary B cells showed a significant negative correlation with neutrophil infiltration”. The negative correlation of neutrophils and B cells was again depicted in neutrophil depletion experiments in Fig. 5. Authors have not provided much explanation on this; it will be good to discuss this aspect in detail and its impact on TB pathogenesis.

[A3]: We observed that in *Nox2^-/-^* mice, neutrophil depletion resulted in the restoration of pulmonary T cells and B cells. However, such restoration was not observed in WT mice. We hypothesized that this discrepancy may be attributed to the nature of *Nox2^-/-^* mice, where excessive neutrophil infiltration could potentially lead to the loss of lymphocytes. Conversely, in WT mice, which did not exhibit neutrophilia, the observed ‘restoration’ may not have occurred because there were already abundant pulmonary lymphocytes.

4. For immature neutrophils I would suggest including Evrard et al., 2018 Immunity article as reference; This was one of the first seminal paper to characterize different neutrophil subtypes.

[A4]: Thank you for your sincere and valuable comments on dissecting neutrophil phenotypes. We have referenced the source and added it to the reference section.

5. Authors mentioned that “CXCR2^lo^CD62L^lo^ immature lung neutrophils were phenotypically heterogeneous from CXCR2^hi^CD62L^hi^ mature lung neutrophils”. This is not clear. Which figure panel shows this? Was any sub-gating of these immature and mature neutrophils (using other markers) was performed? How the heterogeneity was measured / assessed?

[A5]: I apologize for any confusion caused by the use of inappropriate vocabulary. We aimed to highlight the differences in size and granularity of immature neutrophils, as indicated by variations in forward scatter (FSC-A) and side scatter (SSC-A) values, in figure 3D. However, our initial terminology led to confusion. I have revised the content in the manuscript section to avoid further misunderstandings.

6. Fig 5E/6H – Not sure what kind of information tsne plots are adding in this panel. Same goes for other figures where tsne plots have been shown. For example in Fig 6H - I can see many green colour clusters (all are T cells), but what is what; which is CD4, CD8, MAIT, etc etc. If the authors do not have that granular information, avoid including tsne plots. This will be very confusing for the readers.

[A6]: As we did not utilize specific markers for dissecting each immune cell population, such as CD4 or CD8, we were unable to analyze each cluster in detail. We excluded t-SNE plots for figures 6 and 7 to avoid potential confusion. However, we aimed to include t-SNE plots for figures 2 and 6, as they significantly enhance the presentation of key findings, particularly the pronounced infiltration of neutrophils into the lungs.

7. Fig 6 – Does AM80 treatment also ameliorate TB pathology / bacterial load in wild type mice? Why this experiment was performed? Good to make it more clear.

[A7]: We have included data on the impact of AM80 treatment on WT mice in Figure 6. The results suggest that AM80 treatment led to a modest reduction in TB pathogenesis in WT mice, although not as significantly as observed in *Nox2^-/-^* mice. Our decision to administer AM80 treatment to both WT and *Nox2^-/-^* mice *in vivo* was based on its potential to reduce immature neutrophil levels. We underscored its significance by highlighting its effectiveness in patients with leukemia, where it facilitates the generation of mature neutrophils.

 AM80 administration significantly reduced mycobacterial load in the lungs and spleens (Fig 6B) and alleviated lung inflammation (Fig 6C) in male *Nox2^-/-^* mice, However, despite AM80 treatment leading to a reduction in pulmonary bacterial load, its effects were not prominent in male WT mice.

8. Fig S5 – I could not get whether this experiment (depletion of IFN-g and IL-17A) was performed with male or female mice. In the text its mentioned male NOX2KO mice however in the Fig S5 legend its mentioned female NOX2KO mice.

[A8]: I apologize for causing confusions. The experiments described in Supplementary Figure 5 were conducted with female *Nox2^-/-^* mice, as these interventions were found to be highly lethal in male *Nox2^-/-^* mice. We have appropriately revised the manuscript text to reflect this.

*In vivo* neutralization of IFN-γ or IL-17A increased mycobacterial burden, lung inflammation, pro-inflammatory cytokine levels, and pulmonary influx of immature neutrophils in female *Nox2^-/-^* mice.

9. Does depleting G-CSF in wild type mice also has an effect on lung immature neutrophils, bacillary load and pathology? This is very important to investigate as this will generalize the pathological role of immature neutrophils in TB; In my view this study will then have a more clinical relevance.

[A9]: We observed that G-CSF neutralization also decreases the number of immature neutrophils in WT mice. However, no significant differences in bacterial load and lung inflammation were noted after G-CSF neutralization in WT mice. Therefore, we hypothesized that the generation of immature neutrophils driven by G-CSF is a phenomenon specific to *Nox2^-/-^* mice, likely due to excessive production of G-CSF in response to Mtb challenge.

While G-CSF neutralization did not alleviate TB progression in WT mice, it significantly reduced mycobacterial load in the lungs and spleens (Fig 7B) and mitigated lung inflammation (Fig 7C) of male *Nox2^-/-^* mice.

10. Figure legend – please include how many times each experiment was repeated.

[A10]: We added the number of repetitions on each figure legends.

11. Statistical analysis – I am surprised to see that authors have used Mann-Whitney test, though they have more than 2 groups in maximum experimental settings. This is not correct. Whenever there are more than 2 groups ANOVA should be used.

[A11]: Thank you for your guidance regarding this critical point. We have utilized the ANOVA test and made appropriate edits to the figure legends.

12. Depletion experiments - I could not find the info (neither in method section nor in figure legend) on the control Ab used in all depletion studies. Were the control mice receiving any antibody?

[A11]: We incorporated both non-treated mice and control antibody-treated mice in all depletion studies. Our findings revealed that the control antibodies did not exhibit any differences in TB pathogenesis. This information has been included in the Materials and Methods section.

Rat IgG2a, rat IgG1, Armenian hamster IgG, and mouse IgG1 isotype control antibodies were procured from Bio X Cell (West Lebanon, NH, USA). Isotype control antibodies were administered to control mice in correspondence with the original antibodies.

Part III – Minor Issues: Editorial and Data Presentation Modifications

Reviewer #1: The sex differences in NOX2 deficiency are very interesting. However, it may appear that there could be a difference in female mice as well, potentially with lung CFU and neutrophil number. It is not clear from the plots or text if a statistical test has been applied to these differences, which should be addressed on the figures and in the text as appropriate. Related to this: the plots for CFU and inflamed area should include points for individual mice, as there are in every other figure panel.

[A1]: Thank you very much for your appreciation and attention. We included differences between WT and female *Nox2^-/-^* mice, demonstrating that NOX2 deficiency exacerbates TB progression in female mice as well. Additionally, we have modified the plots for CFUs and inflamed area to include individual data points for each mouse.

This indicates NOX2 deficiency plays a significant role in accelerating TB immunopathogenesis, as both male and female *Nox2^-/-^* mice feature exacerbated TB progression. Furthermore, we also found that male sex additionally promotes TB pathogenesis, as indicated by severer TB progression in male *Nox2^-/-^* mice.

Is the same clone of antibody used to deplete neutrophils as it is to detect neutrophils in figures 4 and 5? If so, it is important to see a method of neutrophil quantification that does not rely on Ly6G. This may be able to be accomplished with existing flow markers. As a minor note in this figure, it is a circular argument to say that neutrophil depletion restores the balance between neutrophils and lymphocytes. What does it do to the absolute numbers of lymphocytes?

[A2]: The data presented in Figure 4 and Figure 5 stem from the same experiment. We administered Ly6G-specific neutrophil depletion via intraperitoneal injection, which effectively reduces circulating neutrophil counts but may not completely suppress pulmonary neutrophil accumulation. To achieve comprehensive depletion of lung neutrophils, a combination of intraperitoneal and intratracheal injections of Ly6G-specific antibodies is necessary. This could explain the presence of remaining neutrophils observed in Figure 5A. The increased number of neutrophils, almost one log higher in *Nox2^-/-^* mice, is hypothesized to contribute to the differential mycobacterial CFUs observed in WT and *Nox2^-/-^* mice undergoing neutrophil depletion.

[A3]: Figure 5 demonstrates that neutrophil depletion restores the neutrophil-lymphocyte balance in *Nox2^-/-^* mice, as evidenced by increased percentages and absolute numbers of T cells and B cells.

Would the authors address why there is so much IFNg, IL1, IL6, and IL17 detected in the setting of the respective cytokine depletion experiments? Was there another method implemented to show that neutralization was effective?

[A4]: In order to confirm if each cytokine is properly neutralized, we confirmed the amount of each cytokine in the sera of mice before analyzing immune profiles. The reason there were so much IFN-g, IL-1, IL-6, and IL-17A even after neutralization is likely due to the calculation method, as we calculated cytokine expression from lung lysates. Intrapretoneal injection might be effective for neutralizing circulating cytokines, but they may not have been suitable for perfectly depleting cytokines residing in the lung, and consistently being generated from immature *Nox2^-/-^* neutrophils. Moreover, as we did not neutralize each cytokine at the exact day of sacrifice, few days would have been enough for lung-residing immature neutrophils to generate inflammatory cytokines.

G-CSF is a nice way addition to specifically target neutrophils. It can have multiple effects in addition to granulopoiesis, including on mature neutrophil function and migration into tissues. Evaluating these mechanisms may be outside of the scope of this study, however the authors should address that it might not be as simple as they propose. Also to the above point, did the authors perfuse lungs to ensure that most cells evaluated were within the lung parenchyma? It does not appear that intravascular antibody labeling was completed.
[A5]: Thank you very much for suggesting future milestones for our studies. As we continue to conduct research on neutrophil-mediated TB pathogenesis, controlling pulmonary neutrophil infiltration into the lungs by modulating neutrophil-attracting chemokines could be explored in further studies.

[A6]: We perfused each lung before assessing flow cytometry analysis, and did not label intravascular antibodies.

The mouse lungs were perfused before undergoing flow cytometry analysis.

Displaying cell proportions as pie charts makes is impossible to evaluate statistical relationships. Please include statistical measurements for key comparisons.

[A7]: We mostly dissected neutrophils, T cells, and B cells, which are major immune cells in the lungs of Mtb infected mice. Statically analysis between these cell populations, in terms of immune cell percentages and counts are already attached in each figure, so we did not repeat statistical analysis on pie charts.

When the labels of the graphs are at the bottom require a lot of scrolling to evaluate all panels. Please include labels under each graph.

[A8]: We included labels on the upper parts of each figure and added labels for each FACS plot for convenience.

The key for supplemental figure S6 is missing the anti-IL6 only group.

[A9]: We added label for IL-6 neutralized group in figure S6.

Reviewer #2: (No Response)

Reviewer #3:

1. Antibodies – Please provide the clone information.

[A1]: The clone information is added up in the materials and methods section.

From two weeks post-infection, 250 μg/mouse of anti-Ly6G mAb (clone: 1A8, Bio X Cell, West Lebanon, NH, USA), 200 μg/mouse of anti-IFN-γ mAb (clone: H22, Bio X Cell, West Lebanon, NH, USA), and 250 μg/mouse of anti-IL-17A mAb (clone: 17F3, Bio X Cell, West Lebanon, NH, USA) mAb (Bio X Cell, West Lebanon, NH, USA) were intraperitoneally administered. The antibodies were diluted in PBS and administered three times a week for a total duration of two weeks. From one-week post-infection, 30 μg/mouse of anti-G-CSF mAb (clone: #67604, R&D Systems, Minneapolis, MN, USA), 200 μg/mouse of anti-IL-1R mAb (clone: JAMA-147, Bio X Cell, West Lebanon, NH, USA), 400 μg/mouse of anti-IL-6 mAb (clone: MP5-20F3, Bio X Cell, West Lebanon, NH, USA), 200 μg/mouse of anti-IL-1α mAb (clone: ALF-161, Bio X Cell, West Lebanon, NH, USA), and 200 μg/mouse of anti-IL-1β mAb (clone: B122, Bio X Cell, West Lebanon, NH, USA) (Bio X Cell, West Lebanon, NH, USA) were intraperitoneally administered. The antibodies were diluted in PBS and administered three times a week for a total duration of three weeks. Rat IgG2a, rat IgG1, Armenian hamster IgG, and mouse IgG1 isotype control antibodies were procured from Bio X Cell (West Lebanon, NH, USA). Isotype control antibodies were administered to control mice in correspondence with the original antibodies. From one-week post-infection, 20 μg/mouse of AM80 (Sigma-Aldrich, St. Louis, MO, USA) was orally administered. AM80 was diluted in PBS and administered three times a week for a total duration of three weeks.

2. This article needs an English editing.

[A2]: We have undergone additional English editing for excluding grammatical errors.

3. Enumerating CFU in isolated neutrophils – Did the cells were counted after MACS sorting?

[A3]: Pulmonary neutrophils were enumerated following MACs sorting. The permissiveness of WT and NOX2KO neutrophils was determined subsequent to counting, whereby 10^6^ neutrophils from each group were seeded to mitigate the influence of varying neutrophil numbers.

4. References – Some of the references are not needed can be removed, for example 47 and 49. There are other examples in the text.

[A4]: We excluded each figures from the manuscript text.

5. The way four groups are depicted in Fig 3B is very confusing. Please relabel them.

[A5]: I apologize for causing confusions. We revised Figure 3 to enhance clarity. Figure 3B now focuses on illustrating our gating strategy for distinguishing between mature and immature neutrophils, while Figure 3C specifically discusses the proportions of mature and immature neutrophils in WT and NOX2 deficient mice.
