# Supplementary material for: Permissive lung neutrophils facilitate tuberculosis immunopathogenesis in male phagocyte NADPH oxidase-deficient mice
Source: PLoS Pathog. 2024 Aug 23;20(8):e1012500. doi: 10.1371/journal.ppat.1012500 (PMC11376565; doi:10.1371/journal.ppat.1012500)
Supplement: Attachment — Submitted filename: Choi ES et al._response to reviewers_Second Revision.docx [file ppat.1012500.s013.docx]

Part I - Summary

Reviewer #1: Choi et al. have done a nice job generating and providing additional data, and making progress in addressing the reviewers’ comments in the following areas. First, the analysis of the permissiveness of immature neutrophils using a fluorescent Mtb strain is valuable and supports the authors’ conclusions. Second, showing the effects of G-CSF and AM80 in WT mice, and other necessary comparisons such as analysis of uninfected mice is a very welcome addition. Lastly, the figure clarity and readability has improved.

However, this reviewer still has substantial concerns about many of the conclusions reached by the authors in this manuscript. The article does highlight a major role for neutrophil-driven pathology during Mtb infection in the setting of NOX2 deficiency. However, it is unlikely that this is the only process affected by NOX2 deficiency, and non-neutrophil alternative explanations are only superficially addressed / dismissed. Additionally, the AM80 treatment results in WT mice, together with other data presented in the paper, call into question the interpretation that this treatment acts primarily through neutrophils. Lastly, the fact that findings were observed at high doses with a highly virulent strain, when others have found different results with different strains, could either suggest interesting biology and/or limit the broad applicability of these findings. Given this, the article as written needs some reframing and significant revision of the conclusions drawn from these studies.

Response: We appreciate your valuable comments on the first revision. Thanks to your additional comments and discussions, we were able to improve the study and highlight the role of pulmonary neutrophils in the pathogenesis of TB in male *Nox2^-/-^* mice. We went through additional analysis on WT and *Nox2^-/-^* mice infected with YFP positive Mtb strain, and proposed that immature neutrophils were the major phagocytes featuring mycobacterial permissiveness. We also discussed the possible roles of AM80 treatment in modulating T cell responses or phagocytic capacities of macrophages in *Nox2^-/-^* mouse model, suggesting the need for further, more in-depth studies. Lastly, we discussed our outcomes using male *Nox2^-/-^* mice and the highly virulent Mtb K strain, compared to those infected with H37Rv strain. We concluded that different infection models, such as variations in Mtb strain, initial CFUs, and mouse ages, affect TB pathogenesis across different groups. Again, we deeply appreciate your sincere revision on concluding our manuscript.

Reviewer #2: In this revised manuscript by Choi et al, the authors examine an increase in CFU in male Nox2 mice at 4 weeks post infection and if blocking G-CSF can prevent the neutropenia seen in these animals. The revised manuscript aims to address reviewer concerns, and some of the data are informative, including the YFP-Mtb and the AM80 BMDM experiments and the topically is of great general interest and finding that G-CSF blockade may help block deleterious neutrophil influx is important. However, some of the data provided undercut key conclusions the authors are making in the discussion (and figure legends). These include conclusions around the antimicrobial role of Nox2 in mice and the durability of this CFU difference versus the inflammatory difference. These issues ultimately limit the scope of the paper and limit several of the key conclusions.

Response: Thank you for the detailed review of our revised manuscript. As you noted, we further examined the time-point-dependent TB susceptibility in male *Nox2^-/-^* mice. Building on the initial revision, which focused on the susceptibility in female *Nox2^-/-^* mice, we demonstrate that male *Nox2^-/-^* mice also exhibit elevated mycobacterial CFU and increased pulmonary neutrophil loads at four weeks post-infection. Additionally, we confirmed that the rise in neutrophils began between two to three weeks post-infection, with no significant increase in pulmonary neutrophil loads observed until two weeks post-infection. We also assessed the effect of IL-1R blockade in male *Nox2^-/-^* mice. This approach exacerbated TB pathogenesis and neutrophilia in male *Nox2^-/-^* mice, suggesting that G-CSF, rather than IL-1 signaling, is the primary regulator of immature neutrophil-mediated TB pathogenesis in male *Nox2^-/-^* mouse model. We appreciate your insights once again, which have greatly contributed to the conclusions of our manuscript.

Reviewer #3: In the revised version Choi and colleagues have performed additional experiments and re-analyzed their data (with ANOVA) to support their conclusion and respond to not only my comments (example: adding new data on bone marrow and lung neutrophils profiling in uninfected KO and WT mice, AMD treatment of infected WT mice), but other reviewer comments as well. Together this new data has improved the manuscript. I am particularly excited to see the data of an in vivo experiment (Fig S5) where authors have infected WT and KO mice by YFP+ Mtb and analyzed the uptake of the florescent bacteria by mature and immature neutrophils. They showed (by looking at YFP MFI) the increased permissiveness of immature neutrophils compared to mature one.

We appreciate your sincere and detailed reviews of the manuscript. As previously noted by other reviewers, we incorporated new data on the immunopathogenesis of TB in WT and *Nox2^-/-^* mice by conducting *in vivo* experiments using YFP-expressing Mtb and time point-dependent analysis. We also revised the data by including information on neutrophil properties in naïve mice and correcting the use of statistical analysis. All your comments and advice were extremely valuable for finalizing our manuscript.

Part II – Major Issues: Key Experiments Required for Acceptance

Reviewer #1:

1. This reviewer appreciates the inclusion of the experiment using fluorescently labeled bacteria to demonstrate the increased permissiveness in immature neutrophils. Given the caveats introduced by the differences in bacterial burden at this timepoint, additional analysis is required to support the claim that immature neutrophils are more permissive in the setting of NOX2 deficiency (or if there is increased MFI simply because the bacterial burden in the lung is increased). Looking at additional myeloid populations, such as alveolar macrophages and recruited macrophages, and comparing the fold changes in percent infected cells and YFP MFI in these populations as compared to immature neutrophils in WT and KO mice would give insight into these differences. Additionally, this may give some insight into the roles of macrophages during Mtb infection in NOX2 deficiency.

[A1]: We appreciate your critical comments on the revised data. Regarding your comments on comparing YFP-positive pulmonary immune cells, we reanalyzed our method for enumerating YFP MFI in immune cells. To more accurately compare mycobacterial permissiveness, we concatenated each type of immune cell in equivalent numbers (for instance, 1,000 neutrophils each) and compared the MFI values of each population. Thus, we found that immature neutrophils featured the highest MFI values compared to mature neutrophils, alveolar macrophages, and recruited macrophages in YFP^+^ cells of Mtb-infected male *Nox2^-/-^* mice. Additionally, we documented the fold increases in YFP^+^ alveolar macrophages and recruited macrophages. Despite a significant increase in YFP^+^ macrophage populations in male *Nox2^-/-^* mice, we demonstrated that immature neutrophils remain the most dominant immune cell type among YFP^+^ lung cells and are the most permissive, as indicated by their YFP MFI. We concluded that *Nox2^-/-^* alveolar macrophages and macrophages may also contribute to the increase of pulmonary bacterial load in the male *Nox2^-/-^* mouse model. However, the influx of immature neutrophils is considered to be a more dominant factor, as these cells are predominant in number, exhibit enhanced permissiveness, and are responsible for uncontrolled inflammation. We have reflected all relevant data and discussion in the revised manuscript (**S6 Fig and Page 9-10, Line 248-265)**.

2. The time course is valuable information to have. However, this time course is in female mice and as the authors state, a major advance of the paper is sex differences in NOX2 deficiency, and the major conclusions of the paper are in male mice. This reviewer realizes that the likely reason this data was shown is that the experiment was already done, but I wonder if the results would be the same in male mice. This is not necessarily required to do, but a mention of this time course being done in female mice and the rationale should be called out in the text, not in a supplemental figure legend where it is hard to find. On a related note, from this time course (albeit in female mice) it appears that week 3 is a very interesting timepoint, since differences in neutrophils are already apparent at a time there is similar CFU between KO and WT mice. This would appear to support the authors’ main conclusion of the paper.

[A2]: Your insightful comments prompted us to confirm the time-course-dependent immunopathogenesis of TB in different mouse models. We added new data indicating that four weeks post-infection is also a critical time point for the exacerbation of TB in male *Nox2^-/-^* mice. Time-course-dependent changes in pulmonary CFUs and neutrophil counts were incorporated into main Figure 2. Moreover, we highlighted that the pathological disparity between WT and *Nox2^-/-^* mice, represented by CFUs and neutrophil counts, started increasing between two to three weeks post-infection and peaked at four weeks post-infection time point. Importantly, the increase in neutrophils between WT and *Nox2^-/-^* mice at three weeks post-infection was more significant than that of CFUs. This observation is particularly significant as it suggests that the rise in pulmonary neutrophils may accelerate TB pathogenesis in *Nox2^-/-^* mice by providing an inflammatory and permissive niche for Mtb (**Fig 2E and Page 7-8, Line 201-207. Page 14, Line 393-397)**.

3. How do the authors reconcile the new results shown of AM80 treatment in WT mice, which had an effect, and anti-G-CSF in WT mice, which did not have an effect? The model proposed is that both primarily mediate their effects through decreasing the numbers of immature neutrophils. If this were true, wouldn’t similar effects from both treatments be expected in WT mice? Was macrophage activation in vivo evaluated during treatment, or just in vitro data? Lastly, the conclusion that T cell responses weren’t affected has the caveat that there are significant differences in bacterial burden at the timepoint evaluated. Couldn’t AM80 have induced an earlier/stronger Th1 response, which is diminished at week 4 due to decreased CFU? Given these caveats, and the strength of the G-CSF results, this reviewer wonders whether this story would be stronger without the inclusion of this experiment.

[A3]: Thank you very much for your detailed discussion of our data. We concluded that G-CSF neutralization showed therapeutic potential only in male *Nox2^-/-^* mouse model, characterized by excessive G-CSF production due to mycobacterial infection. As WT mice do not exhibit such uncontrolled expression of G-CSF, neutralizing G-CSF did not impact TB pathogenesis in these mice. In addition, AM80 modulates myeloid cell production through G-CSF independent pathways [1]. We discussed the possible reasons why AM80 featured therapeutic potential in both mouse strains, while G-CSF neutralization was specifically effective in mitigating TB in male *Nox2^-/-^* mouse model (**Page 16, Line 444-453)**.

Although we did not investigate the functional properties of macrophages in AM80-treated male *Nox2^-/-^* mice, treating bone marrow-derived macrophages (BMDMs) with AM80 showed no differences in bacterial clearance. However, considering your insightful comments and the multifunctional immune alterations induced by AM80, it is reasonable to hypothesize that AM80 may also modify macrophage functions *in vivo*. We also value your comments on T cell responses. Since we did not evaluate Th1 responses at two or three weeks post-infection, it is plausible to hypothesize that AM80 could enhance Th1 responses and reduce mycobacterial levels during these early stages. Your comments were incredibly valuable for further exploration of AM80's impact on anti-TB immunity, particularly in studies focusing on both T cell responses and macrophage functions (**Page 17, Line 486-490)**.

[1]	Li L, Qi X, Sun W, Abdel-Azim H, Lou S, Zhu H, et al. Am80-GCSF synergizes myeloid expansion and differentiation to generate functional neutrophils that reduce neutropenia-associated infection and mortality. EMBO Mol Med. 2016;8(11):1340-59. <https://doi.org/10.15252/emmm.201606434> PMID:27737899

Reviewer #2: Major Comments:

1. Central to the conclusions of this paper are the CFU differences in male mice shown generally at 4 weeks. At the request of the reviewers a time course is now shown that shows what has previously been observed, that the small CFU differences are only seen significantly at 4 weeks post-infection. While the authors say 3 weeks is an increase there are no statistics included and the differences look minor. Importantly, while the authors say the difference in neutrophils goes back to normal, these actually seem to persist with no differences in CFU. In line with other previous studies using Nox2 mice. With these data in the revision, the only supported conclusion is that male Nox2 mice have a transiently higher CFU at 4 weeks, but the paper is written to make these differences in CFU a larger conclusion which is not robustly supported by the data shown.

[A1]: Thank you very much for your detailed and critical comments on the revised data. We updated the data on the time-course-dependent TB pathogenesis in female WT and *Nox2^-/-^* mice, adding statistical analyses for CFUs and neutrophil counts at each time point to enhance clarity (**S1A, S1B fig and Page 7, Line 179-182)**. Furthermore, we added a new *in vivo* data illustrating that the increase in lung CFUs and neutrophils in male *Nox2^-/-^* mice occurs in a time-point-dependent manner. These new data emphasize the importance of constant neutrophil accumulation and the immunopathogenesis of TB in male *Nox2^-/-^* mouse model (**Fig 2E and Page 7-8, Line 201-207)**.

2. The inhibition of IL1R included in the rebuttal is also confusing. IL1 has a key role in early antimicrobial responses, so blocking IL1R as early as a week following infection is going to have severe effects, as seen by the authors. It is interesting that the male mice succumbed to disease and couldn’t be used in this experiment, but using females complicates comparisons across experiments. Thus, with the way this experiment was done I’m not convinced the data can really discern what the role of IL1a or IL1b is in this model.

[A2]: Your valuable comments prompted us to redesign our experiment, given the critical role of IL-1 signaling in initiating anti-TB innate immunity. Despite literature from Olive et al. suggesting that IL-1R blockade mitigates TB pathogenesis in *Nox2^-/-^* mice [2], our adaptation of their protocols to start IL-1R blockade from two weeks post-infection in male *Nox2^-/-^* mice had the opposite effect, exacerbating TB pathogenesis at four weeks post-infection. This led to increased pulmonary mycobacterial load, lung inflammation, and neutrophil counts. Therefore, our data reveal that G-CSF, rather than IL-1 signaling, is crucial for initiating immature neutrophil-mediated TB pathogenesis in male *Nox2^-/-^* mice. The disparity between our findings and previous reports is likely due to differences in the infection model system, characterized by variations in mycobacterial strain virulence, initial infectious doses, and the ages of the mice used. In conclusion, while IL-1R blockade may suppress neutrophil-mediated TB pathogenesis in a less severe infectious model, it proved ineffective in our model involving a severe infection with a high dose of the Mtb K strain load (**S10 Fig and Page 12, Line 335-340. Page 17, Line 467-473)**.

[2]	Olive AJ, Smith CM, Kiritsy MC, Sassetti CM. The Phagocyte Oxidase Controls Tolerance to Mycobacterium tuberculosis Infection. J Immunol. 2018;201(6):1705-16. <https://doi.org/10.4049/jimmunol.1800202> PMID:30061198

Part III – Minor Issues: Editorial and Data Presentation Modifications

Reviewer #1:

1. The authors should entertain additional possibilities (such as other cell types playing a role) as to why the neutrophil depletion did not fully mitigate pathology and decrease bacterial burdens to WT mice. Also, why was the IA8 depletion not efficacious in this setting, when others have depleted pulmonary neutrophils during Mtb infection with much greater effect (DOIs: 10.1038/nmicrobiol.2017.72; 10.1172/JCI130546)?

[A1]: Thank you for your detailed comments. According to previous studies, intraperitoneal depletion of neutrophils using anti-Ly6G monoclonal antibodies (mAbs) may not entirely remove neutrophils from the lungs of treated mice. Boivin et al. noted that intraperitoneal injection of anti-GR1 or Ly6G monoclonal antibodies may not fully eliminate neutrophils, as newly synthesized young neutrophils can replenish the blood or organs even after depletion [3]. Given that anti-Ly6G mAbs (1A8) mediate slow neutrophil depletion through Fc-dependent opsonization and phagocytosis of neutrophils by macrophages, and considering that NOX2 deficiency impairs macrophage functions, both excessive neutrophil production and macrophage malfunction due to NOX2 deficiency are considered to be reasons for the incomplete depletion of pulmonary neutrophils in this mouse model (**Page 16, Line 453-461)**.

[3]	Boivin G, Faget J, Ancey PB, Gkasti A, Mussard J, Engblom C, et al. Durable and controlled depletion of neutrophils in mice. Nat Commun. 2020;11(1):2762. <https://doi.org/10.1038/s41467-020-16596-9> PMID:32488020

2. The methods describing quantification of lung inflammation are still lacking in specifics. How was inflammation determined? Was it with a specific plugin in ImageJ? By eye? If the latter, was the scorer blinded? This information is crucial to ensure reproducibility.

[A2]: We appreciate your valuable comments. We have added methodological details on the quantification of pulmonary inflammation in Materials and Methods section of the revised manuscript (**Page 21-22, Line 594-609)**.

3. In instances where there is a significant difference between groups, they should not be concluded to be the same. Specific figures to note are: Fig 1E (IL-10 is significantly upregulated in male KO vs WT); Fig S5A-B (the number of total, immature, and mature neutrophils are slightly increased in naïve NOX2 deficient male mice as compared to WT, please change corresponding test); Fig 6D (TNF significantly decreased with AM80 in WT, perhaps choose another word such as “substantially” here?).

[A3]: I appreciate your detailed comments on the manuscript. As you suggested, we have thoroughly revised the descriptions, including those mentioned in Fig 1E and Fig 6D, to provide more accurate and detailed information for the readers (**Page 7, Line 187-190. Page 10, Line 276-277. Page 11, Line 307-311)**.

Reviewer #2: (No Response)

Reviewer #3: N/A
